# Determining health professional students’ self‐perceived cultural capability following participation in clinical placement with Aboriginal and Torres Strait Islander Peoples: A systematic review

**DOI:** 10.1002/jfa2.70017

**Published:** 2024-12-09

**Authors:** Kate Paisley, Sean Sadler, Matthew West (Wiradjuri), James Gerrard, Rhonda Wilson (Wiradjuri), Angela Searle, Vivienne Chuter

**Affiliations:** ^1^ Discipline of Podiatry University of Newcastle Darkinjung (Ourimbah) New South Wales Australia; ^2^ Discipline of Podiatry School of Health Science Western Sydney University Dharawal (Campbelltown) New South Wales Australia; ^3^ Central Australian Aboriginal Congress Mparntwe (Alice Springs) Northern Territory Australia; ^4^ School of Nursing and Midwifery University of Newcastle Darkinjung (Gosford) New South Wales Australia; ^5^ School of Nursing Massey University Palmerston North Aotearoa (New Zealand); ^6^ Discipline of Nursing School of Health and Biomedical Sciences RMIT University Naarm (Melbourne) Victoria Australia

**Keywords:** Aboriginal and Torres Strait Islander, clinical placement, cultural capability, systematic review

## Abstract

**Background:**

Collective evaluation of studies assessing students’ self‐perceived cultural capability following clinical placement is required to help inform future cultural capability training for both university and healthcare service environments. Therefore, the aim of this systematic review was to evaluate studies investigating health professional students’ self‐perceived cultural capability following participation in a clinical placement with First Nations Peoples.

**Methods:**

Electronic database searchers were conducted in MEDLINE, EMBASE, AMED, PsychINFO, Pubmed, CINAHL and Informit. Hand Searches of grey literature were conducted including Lowitja institute, Australian Indigenous HealthInfoNet, Menzies School of Health Research, Services for Australian Rural and Remote Allied Health, and the Australian Institute of Health and Welfare. Studies published in English that investigated health professional students’ self‐perceived cultural capability before and after clinical placement undertaken with First Nations people in Australia were eligible for inclusion. Two authors independently screened potentially eligible studies and performed quality appraisal and data extraction.

**Results:**

A total of 14 studies were included (*n* = 307 participants). Studies included undergraduate students from podiatry, medicine, nursing, pharmacy, and mixed health professions. The results of this systematic review suggest that clinical placements in health services or settings for Aboriginal and Torres Strait Islander Peoples that involve elements of co‐design are effective in increasing aspects of health professional students’ self‐perceived cultural capability. This outcome was consistent across studies regardless of the location of clinical placements (urban or rural), type of clinical placement (health setting or Community), or length of placement.

**Conclusions:**

The findings from this systematic review suggest that clinical placement in health services or settings for Aboriginal and Torres Strait Islander Peoples may contribute to increased self‐perceived cultural capability in health professions graduates. However, the impact of the placements on the cultural safety of student‐led care, from a First Nations perspective, remains to be established.

## 1 BACKGROUND

The ongoing impact of colonisation in Australia has seen the enduring racism, disadvantage and trauma, political exclusion and loss of First Nations health paradigms that were evolved from what is the world’s oldest living culture [1, 2, 3, 4]. Consequently, First Nations people have a lower life expectancy, higher likelihood of hospitalisation and poorer health outcomes compared to non‐Indigenous Australians [5]. These disparities are perpetuated by lack of access to culturally safe care with a quarter of Aboriginal and Torres Strait Islander people reporting unsafe care experiences every year [6].

The National Scheme’s Aboriginal and Torres Strait Islander Health and Cultural Safety Strategy 2020‐2025 (the Strategy) developed in close partnership with Aboriginal and Torres Strait Islander organisations and released by the Australian Health Practitioners Regulation Agency (Ahpra) aims to achieve health equity by 2031 through provision of a culturally safe health environment [7]. Fundamental to overcoming current inequities in health care access, and to delivery of culturally safe care, is a culturally capable health workforce [8]. Cultural capability refers to the skills, knowledge and behaviours required to plan, support, improve and deliver clinical healthcare services in a culturally respectful and appropriate manner [9]. The requirement for provision of culturally safe care to First Nations people in Australia is reflected across health professions in profession‐specific capabilities and their accreditation requirements [7, 8].

The Aboriginal and Torres Strait Islander Health Curriculum Framework (The Framework) supports higher education providers to implement Aboriginal and Torres Strait Islander health curricula across their health professional programs [10]. However, this implementation is not mandated nationally which allows higher education providers to select any form of cultural capability training to enable it to be tailored to individual Communities across the First Nations [10]. As a result, across Australian tertiary educational institutions, there are heterogeneous pedagogical approaches to cultural capability training including online and face‐to‐face learning modules, participation in discrete courses and workshops, and clinical placement [10].

Several studies evaluating cultural capability training have found health professions students demonstrate improved understanding of, and attitudes towards, Aboriginal and Torres Strait Islander health and self‐perceived preparedness for practising in Indigenous Communities following clinical placement experiences [11, 12]. However, previous systematic evaluation of studies are either not current, exclude one‐off or short‐term clinical experiences [13], or they are focused specifically on curricula development [14]. Collective evaluation of studies assessing students’ self‐perceived cultural capability following clinical placement is required to help inform future cultural capability training for both university and healthcare service environments. Therefore, the aim of this systematic review is to determine health professional students’ self‐perceived cultural capability following participation in clinical placement involving health care provision to First Nations people.

## 2 METHODS

This systematic review was prospectively registered with PROSPERO (CRD42021277102) and has been reported in accordance with the PRISMA checklist [15].

### 2.1 Search strategy

Medline, Embase, Amed, Psychinfo, Pubmed, Cinahl and Informit databases were searched from inception to February 2024 (Supplementary File 1). Hand searches were conducted through the Lowitja Institute, Australian Indigenous HealthInfoNet (www.healthinfonet.ecu.edu.au), Menzies School of Health Research, Services for Australian Rural and Remote Allied Health (www.sarrah.org.au), and the Australia Institute of Health and Welfare (http://www.aihw.gov.au/).

### 2.2 Eligibility criteria

Any qualitative or quantitative original study conducted in Australia and published in English that investigated students’ perceptions of their own cultural capability following clinical placement involving provision of health care with and for First Nations people was eligible. Due to the variable terminology to describe the intent and outcomes of these placements (e.g., cultural capability, cultural responsiveness, cultural awareness) studies reporting outcomes relating to the skills, knowledge and behaviours required to plan, support, improve and deliver clinical healthcare services in a culturally respectful and appropriate manner were included. Studies had to evaluate cultural capability before and after the placement and include undergraduate or postgraduate health professional students. Clinical placement sessions were broadly defined to include student interactions with First Nations people in a hospital, health clinic, or Community setting. No restriction was applied to the duration of the clinical placement. Studies that included clinical placements that did not specifically involve Aboriginal and/or Torres Strait Islander people were excluded. Studies that evaluated a range of different cultural capability training methods in addition to clinical placement, for example tutorials or workshops, were eligible.

### 2.3 Study selection and data extraction

Two authors (KP and SS) screened retrieved articles at title, abstract, and full text level. Data were independently extracted by one author (KP), using a customized data extraction form, and cross‐checked by two authors (SS and AS). Data extracted includes: study, participant, and cultural capability training characteristics, and students’ perceptions of the cultural capability training.

### 2.4 Quality appraisal

The Aboriginal and Torres Strait Islander quality appraisal tool (QAT) was used to appraise studies including assessing for Aboriginal and Torres Strait Islander values and principles for ethical research and therefore the cultural safety of the study [16]. Considering potential restrictions related to journal formatting requirements that have historically created barriers to reporting details required as part of the Aboriginal and Torres Strait Islander QAT, included articles that were related (for example relating to the same clinic) were also checked for information where possible. Assessment was performed by a First Nations (MW or RW) and non‐Indigenous researcher (VC). For articles where these reviewers were conflicted due to authorship a fourth reviewer (non‐Indigenous) undertook this task (JG). Secondly, methodological quality appraisal and risk of bias were assessed using the observational study appraisal checklist which was independently performed by two researchers (KP and AS) [17]. Disagreements were arbitrated by a third reviewer (SS).

## 3 RESULTS

**FIGURE 1:**
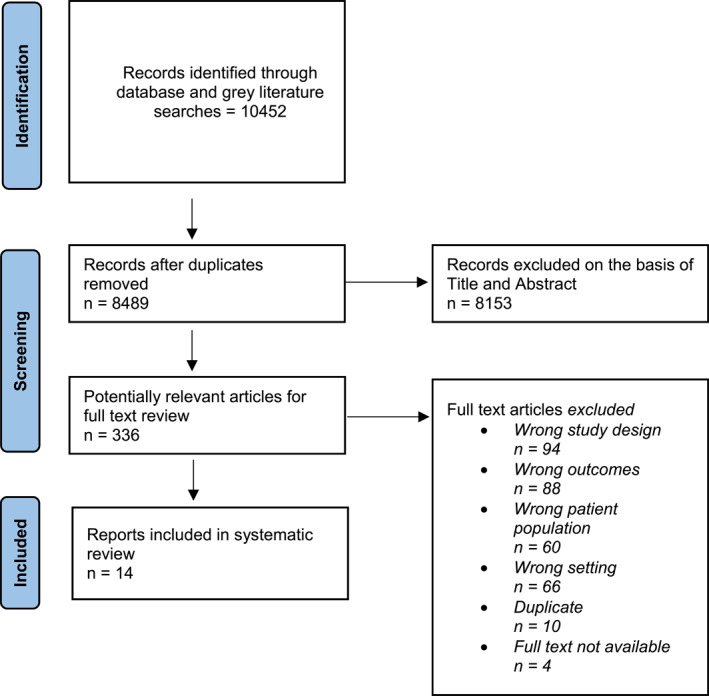
PRISMA flow diagram.

A total of 336 studies were appropriate for full review after which 14 were included (Figure 1) (Supplementary File 1).

### 3.1 Characteristics of included studies

Of the 14 studies included (*n* = 307 participants), four were quantitative [12, 18, 19, 20], five were qualitative [21, 22, 23, 24, 25], and five employed a mixed‐methods approach in study design [26, 27, 28, 29, 30] (Table 1). Across the 14 studies, a total of 307 participants were included with cohorts from New South Wales (*n* = 4), Western Australia (*n* = 3), Northern Territory (*n* = 2), Queensland (*n* = 1), South Australia (*n* = 1) and three studies not reporting their location [24, 27, 30]. Studies included undergraduate students from medicine [12, 18, 19, 21, 22, 27, 30], nursing [24, 26, 29], pharmacy [28], podiatry [20], as well as a combination of multiple disciplines including speech pathology and occupational therapy [25], and dietetics, health promotion, human communication science, medicine, nursing, occupational therapy, pharmacy, and physiotherapy [23].

**TABLE 1:** Overview of Included studies.

Author, Year, Location	Discipline/Level of study	Placement	Students, n, mean age (SD), gender	Pre‐placement training
Askew et al. 2017 [21] Brisbane Queensland	Medicine Undergraduate and medical registrars	4 weeks to 12 months clinical placement at an urban Aboriginal and Torres Strait Islander primary healthcare service.	*N* = 11 (7—students, 4—registrars, Mean age = 30 Gender = 50% female	Not reported
Bennett et al. 2013 [26] New South Wales	Nursing Undergraduate	4 to 8 weeks clinical placement in rural and remote sites across far west New South Wales, including mainstream primary healthcare services or Aboriginal Community Controlled Health Organisations.	*N* = 31 Mean age = 30 Gender = 84% female	5 day orientation that includes cultural education, primary healthcare theory and practice, Community integration, resilience, person centered care, and rural and remote practice presented by Indigenous and non‐Indigenous academics and support by host site clinicians.
Benson et al. 2015 [27] Not Reported	Medicine Undergraduate	2 day trip to a remote Aboriginal Community of 250 people with activities including supervision at the local community pool, assisting with medical treatments, undertaking research on scabies, pharmacy audits and delivering food to elderly.	*N* = 23 Mean age = 23 Gender = 50% female	Cultural awareness training as part of university studies. Aboriginal Health Workers provided cultural orientation.
Bird et al. 2022 [25] East Arnhem, Northern Territory	Allied Health Undergraduate (final year)	8 week clinical placement undertaken in Nhulunbuy and Yirrkala and surrounding Aboriginal Communities of the East Arnhem region of the Northern Territory providing a service for older Yolnu and younger Yolnu with a disability.	*N* = 4 (2 occupational and 2 speech pathology students) Mean age = 22.5 Gender = 100% female	Week 1 of placement students participated in orientation to the Community and to Yolnu culture and languages.
Jamrozik, 1995 [18] Western Australia	Medicine Undergraduate (3^rd^ year)	8 day field trip to Aboriginal Communities and local health services in the eastern goldfields region of Western Australia. 2 days of travel. 1 day meeting with Aboriginal people 1 day at local district hospital to interview staff regarding experiences of Aboriginal patients and people. 3 days in Community talking to health staff and residents. 1 day visiting a health centre for mothers and children, the local Aboriginal Medical Service, and a remote Community.	*N* = 9 Mean age = Not reported Gender = Not reported	Preparatory reading/lecture on Aboriginal health delivered by chief Health officer and director of Aboriginal Programs. Accompanied by guide who lived in the area and had relatives in the host Community.
Kamien, 1975 [22] Bourke, New South Wales	Medicine Undergraduate (5^th^/6^th^ year)	2 to 5 weeks clinical placement attached to an Aboriginal Community health research project in Bourke in Western New South Wales. Working with staff at the local hospital, St Johns Ambulance, Community Health, a Service organisation in town, and at clinics in the Aboriginal Community.	*N* = 14 Mean age = Not reported Gender = Not reported	Guided by two Aboriginal field officers and three matriarchs (obtained by personal email from Kamien—23/10/21).
Lockhart et al. 2003 [23] Western Australia	Allied Health Undergraduate (final year)	1 week multidisciplinary rural placement program in a primary health care setting for Aboriginal and Torres Strait Islander People.	*N* = 23 (dietetics, health promotion human communication science, medicine, nursing, occupational therapy, pharmacy and physiotherapy students) Mean age = Not reported Gender = Not reported	Not reported.
Morrissey and ball 2014 [28] Northern Territory	Pharmacy/Clinical Science Undergraduate (1^st^ year)	5 day group placement to health facilities, cultural places and Communities in Katherine, Darwin, and surrounding communities in the Northern Territory.	*N* = 29 Mean age = Not reported Gender = Not reported	Formal cultural awareness training prior to placement departure. Supervisor consultant of Indigenous descent.
Paul et al. 2006 [12] Western Australia	Medicine Undergraduate (final year)	1 year of RCS which provides rural based education at one of eight sites across Western Australia. RCS allows for students to gain practical learning opportunities, as well as clinical placements at local hospitals, general practices, Community and remote clinics, and Aboriginal medical centres.	RCS = N = 15 Mean age = Not reported Gender = Not reported	Aboriginal health orientation seminar 7 to 11 hours of formal teaching and learning in Aboriginal health throughout their course.
Power et al. 2020 [24] Not reported	Nursing Undergraduate (3^rd^ year)	3 week urban clinical placements at sites including an Elders facility, Non‐government Organisations catering to young Aboriginal mothers and a primary healthcare service visiting new mothers and babies at home.	*N* = 8 Mean age = Not reported Gender = Not reported	Aboriginal Community engagement elective replaces lectures with walking tours, visits to Aboriginal Medical Service, assisting NAIDOC celebrations and yarning with Aboriginal Elders. Aboriginal Community engagement elective was written and facilitated by an Aboriginal academic.
Warren et al. 2016 [19] South Australia	Medicine Undergraduate	Outreach trips 1 to 7 days (avg 4 days) accompanying general practitioners, ophthalmologists, allied health professionals, respiratory physician, paediatric cardiologist to clinics in remote Indigenous communities e.g., Ceduna, Yalata, Alice Springs) *n*=20. Clinical placements (remote Indigenous psychiatry terms) 3‐4 weeks (Katherine and Anangu Pitjantjatjara Yankunytjatjara Lands) *n*=4.	*N* = 24 Mean age = Not reported Gender = Not reported	Cultural Communication workshop—2 days. Developed and run by the Yaitya Purruna Indigenous Health Unit at the University of Adelaide.
Webster et al. 2010 [29] Broken Hill New South Wales	Nursing Undergraduate (2^nd^ year)	4 weeks rural placement in Community Health Centres and Aboriginal Community Controlled Health Organisations within far western New South Wales.	*N* = 8 Mean age = 34 Gender = 62.5% female	Not Reported.
West et al. 2021 [20] Wyong New South Wales	Podiatry Undergraduate (final year)	4 days at a culturally responsive Aboriginal and Torres Strait Islander student clinic at Wyong Hospital in New South Wales.	*N* = 58 Mean age = Not reported Gender = 53% female	1 day cultural capability training program delivered by an Aboriginal podiatrist and an Aboriginal Elder from the local Community.
Wright et al. 2014 [30] Not Reported	Medicine Undergraduate	11 days rural clinical or Community placement. While all students received information about Aboriginal healthcare, most did not experience an Aboriginal healthcare placement.	*N* = 50 in total Rural program Mean age = Not reported Gender = Not reported	2 day rural orientation A Cultural Safety Day was held as part of the orientation by an Indigenous team.

Abbreviations: NAIDOC, National Aborigines and Islanders Day Observance Committee; RCS, Rural Clinical school; ±SD, ± one standard deviation.

The majority of studies measured the effects of the placement on students’ self‐perceived cultural capability immediately following placement [12, 18‐21, 23, 24, 27‐30], with only three studies conducting longer term follow‐up up to 2 months [25], at 3 months [26], and 2 months to 2.5 years following the end of the placement [22]. The clinical placement experiences varied widely from a 2 day fly in fly out trip [27] to a 1 year placement [12, 21], with the others offering varied time periods of less than 1 week [19, 20, 28], 1 week to 1 month [18, 23, 24, 29, 30], or greater than 1 month but less than 2 months [22, 25, 26]. The locations of the clinical placements also differed with 11 of the 14 studies taking place in rural and remote locations [12, 18, 19, 22, 23, 25, 26, 27, 28, 29, 30] and the other three in urban locations [20, 21, 24]. The experiences during the placements also varied with some having predominately clinic‐based training [12, 19, 20, 21, 22, 24, 26, 29], others providing Community‐based exposures such as informal visits to cultural places and lunches with Aboriginal and Torres Strait Islander people [23, 28, 31], or a combination of these experiences [18, 25, 27, 30].

The provision of a form of cultural capability training prior to the clinical placement was reported for 11 studies [12, 18, 19, 20, 22, 24, 25, 26, 27, 28, 30] with all but one [12] using a co‐design approach to either develop the training, facilitate and present the training, or accompany students throughout the placements. Pre‐placement training varied in content and is described in Table 1.

### 3.2 Outcomes of included studies

Three studies used surveys and/or questionnaires [12, 18, 20], one used written responses [24] and eleven used a combination of outcomes measures such as survey, semi‐structured interviews, focus groups and written responses [19, 21, 22, 23, 25, 26, 27, 28, 29, 30] (Table 2). Consistent across the studies was the finding that students’ self‐perceived cultural capability improved following clinical placement. Specifically, studies found that students reported a greater awareness and understanding of the interrelationship between culture, family, and Community and how it affects both health, and access and engagement with health care [19, 20, 21, 22, 24, 25, 27, 28, 29, 30]. Additionally, students reported increased confidence with communication and providing culturally safe clinical care for Aboriginal and Torres Strait Islander people following their placement [12, 19, 20, 21, 24, 25, 26, 30].

**TABLE 2:** Summary of Included Study Findings.

Study, year	Outcome measure	Findings
Askew et al. 2017 [21]	Written responses to five questions requiring clinical decisions (summary of patient, actions required, additional information required, preliminary healthcare plan, any assumptions made) pre‐placement. Guided semi‐structured interviews pre‐placement where participants described their imaginings of the patient and the reasoning and assumptions behind their clinical decision making. Semi‐structured interview post‐placement reflecting on initial responses and assumptions.	Themes identified: practitioner confidence and cultural safety; approaches to the practitioner‐patient relationship; and shifting from theoretical knowledge to practical understanding. All participants increased their ability to work in a more humanistic manner, with increased capacity for culturally safe practice. Participants expressed greater recognition of the nuances and complexities inherent in their patients’ Aboriginality. Several participants gained awareness of the effect of past aggressive assimilationist policies on Aboriginal health today. Post‐placement all participants exhibited deeper and more practical understandings of the role of trust in patient engagement.
Bennett et al. 2013 [26]	Survey (confidence log using 5‐point likert scale, 4 questions regarding pre‐practicum perceptions of rural life, Indigenous health, and primary healthcare). Focus group (discussion with prompting/clarifying questions). 3 month post‐placement phone interview.	Question 1 ‐ communicating effectively with Indigenous people: Pre = 35.5%, Post = 62.1%, Increase = 26.6%. Question 2 ‐ providing culturally appropriate care: Pre = 26.7%, Post = 55.1%, Increase = 28.4%. Question 3 ‐ understanding the needs of Indigenous clients: Pre = 13%, Post = 62.1%, Increase = 49.1%. Question 4 ‐ asking Indigenous people questions about their healthcare: Pre = 26%, Post = 66%, Increase = 40%. Increased confidence in interacting with Indigenous people; Increased confidence in primary healthcare. 68% of participants signaled intention to move to rural/remote locations because of this experience. At 3 month follow up 36% of these participants had relocated post‐graduation to rural and remote health facilities or organisations.
Benson et al. 2015 [27]	2 questionnaires pre‐placement (1^st^ on demographic parameters of participants; 2^nd^ on previous experiences in Aboriginal Communities, medical course preparation for the trip and future interest in Aboriginal health) (4‐point likert scale). Pre (expectations) and post (outcomes) short written narratives under the headings: Cultural, Medical, and Personal.	Themes identified: Cultural; Medical; and Personal. For most participants the trip was a ‘cultural eye‐opener’, and they acknowledge that no amount of reading or lectures could have prepared them for it. Students reflected on their experience as a valuable glimpse into the social, cultural, economic, and geographical issues that affect people’s health status. Despite prior learning, none of the students reported feeling prepared for their experience. Post‐placement students felt they had a better grasp of how imperative it is to take a patient’s cultural practices into consideration when planning healthcare management.
Bird et al. 2022 [25]	Semi‐structured interviews occurred over a 2 month timeframe following completion of the placement. Questions focused on the service and value to older Yolnu, the Community and students. Participants were interviewed via zoom videoconferencing by a researcher not known to the participants. The interview guide included questions regarding their involvement and experience with the services, the impact of the service and if they would like to see the services continue. Interview duration ranged from 28 to 83 minutes with an average of 51 minutes.	Themes identified: ‘Learning to connect and connecting to learn’; ‘preparing and supporting’; ‘bonding and responding’; ‘growing and enriching’; and ‘working and weaving’. Students emerged feeling stronger and more able to provide a culturally safe and responsive service. “We have learnt so much in such a short period of time… and we’ve had (an) experience with clients we never thought… we would be able to see.” Students grew in skills, knowledge and confidence and became more comfortable with the challenges associated with working within the remote Community. As bonding increased, students’ understanding of what mattered to the Community grew and expanded. Students were able to provide a person‐centered and culturally responsive service.
Jamrozik 1995 [18]	Survey (5‐point likert scale, 12 statements about Aboriginal people and their situation). 15‐item questionnaire (assessing knowledge of Aboriginal history, health, and culture). Post‐placement questionnaire included 10 further statements about aspects of the trip, plus open‐ended questions about good and less satisfactory features of the experience.	Student responses to factual questions about Aboriginal people increased post‐placement (e.g., proportion of Aboriginal people in the Western Australia population; proportion of Aboriginal people in perth; Aboriginal male life expectancy at birth; proportion of non‐drinkers among: non‐Indigenous men/women, Aboriginal men/women; name behaviors likely to offend Aboriginal patients). Post‐placement the average number of Aboriginal and Torres Strait Islander people that the students had spoken to increased by a factor of 3. Responses to questions about attitudes to Aboriginal and Torres Strait Islander people did not change much pre/post placement. However, large changes seen in attitudes concerning the homogeneity of and anticipated hostility from Aboriginal people.
Kamien 1975 [22]	Pre‐placement letter stating what they expected from their stay before arrival. Post‐placement letter, 2 to 4 months after, expressing views on experience. Meeting with writer 2 months to 2.5 years after placement and comments on their experiences recorded.	Greater understanding of the conditions of the Aboriginal people in the area and began to understand how a group’s culture and norms can affect its way of life. Gained insight into the magnitude of the social and medical problems of Aboriginal people. Main comments from before and after the placement detailing the benefits of the experience: different experience from that found in large hospitals; learning of other facts than those taught at medical school; help in deciding about future career; experience of life in a country town; opportunity to assess the importance of the delivery of healthcare; awareness of the role of paramedical personnel; insight into general practice.
Lockhart et al. 2003 [23]	Pre‐ and Post‐placement surveys (no details provided). Individual and group discussions with students (no details provided).	Most students had little or no experience working with Aboriginal and Torres Strait Islander people and this placement did not provide the cross‐cultural opportunities that students had desired. Greater understanding of difficulties associated with implementing a primary healthcare approach in a setting with a relatively marginalised Aboriginal and Torres Strait Islander population.
Morrissey et al. 2014 [28]	Pre‐ and post‐placement cultural awareness questionnaire. Post‐placement cultural awareness multiple choice quiz. Pre‐ and post‐placement measurements using the modern Racism scale (MRS) and the Attitudes Towards Indigenous Australians (ATIA) scale. Verbal reflections and reflective journals.	Developed greater understanding of aspects in the Indigenous Community including their beliefs and traditions. Empathy towards Aboriginal people (MRS): Pre = 34%, Post = 48%, Increase = 14%. Positive attitude towards Aboriginal people—Impartiality (ATIAS): Pre = 64%, Post = 78%, Increase = 14%. Positive attitude towards Aboriginal people—Empathy (ATIAS): Pre = 52%, Post = 62%, Increase = 10%. Post‐placement Cultural Awareness quiz (% correct answers): 93% ‐ What is one of the skills required to work with Aboriginal people? 100% ‐ What does Aboriginal protocols mean? 86% ‐ in a health setting what does cultural security and cultural respect mean? 100% ‐ list your understanding of what cross cultural awareness is?
Paul et al. 2006 [12]	Preparedness to practice questionnaire (beginning of year 6 and on first day of internship)—2 items asking students to rate how well prepared they were to perform or practice skills and abilities related to Aboriginal health.	RCS interns had a significantly higher perception of their ability to communicate with Aboriginal people and apply knowledge to provide culturally secure care than the urban‐based University of Western Australia graduates. I can communicate appropriately with Aboriginal people: Year 6 (2003): Median score (IQR) = 3 (3‐4); Year 6 (2004): Median score (IQR) = 4 (3‐4); interns (2005): Median score (IQR) = 4 (3‐4), p value = 0.005. I apply knowledge of Aboriginal health to provide culturally secure health care: Year 6 (2003): Median score (IQR) = 3 (3‐3); Year 6 (2004): Median score (IQR) = 4 (3‐4); interns (2005): Median score (IQR) = 4 (3‐4), p value = <0.001.
Power et al. 2020 [24]	Online Journal (before, during and after clinical placement, responding to 6 trigger questions regarding current knowledge of Aboriginal people, clinical placement perceptions and actual experience, experiences interacting with Aboriginal people that have enhanced an understanding of Aboriginal culture or people, building rapport and trust with Aboriginal clients and staff members, and plans for continuing to develop cultural capability to work effectively with Aboriginal people). 2000 word reflective essays made of online journal entries.	Themes: working with inexperience and uncertainty; developing acceptance and understanding; becoming allies and advocates. Evidence of student growth over the duration of their clinical placement through positive experiences with Aboriginal people. Prior to the subject some students had little knowledge of Aboriginal people, particularly international students. Immersion in the Community clinical settings increased insight gained into the wrongs experienced by Aboriginal people. Increased cultural confidence. Value of hearing Aboriginal peoples’ stories firsthand through interacting and yarning personified colonisation and engendered empathy. Students understood there was a need for change and identified that everyone has a role in actively working towards positive change.
Warren et al. 2016 [19]	Online survey pre/post placement (6‐point likert scale of 0–5 and reported as mean value (±SD) and 1 yes/no question). Reflective post trip report.	Q1 How likely are you to work in an Indigenous Community in the future? Pre = 3.2/5 (± 1.1), Post = 4.0/5 (± 0.8), P = 0.006. Question 2 How well prepared do you feel to work in an Indigenous Community in the future? Pre = 1.8/5 (± 0.8), Post = 3.2/5 (± 0.8), P value = <0.001. Question 3 (How likely are you to work in a rural/remote area in the future?): Pre = 3.3/5 (± 1.1), Post = 4.1/5 (± 0.6), P value = 0.002. Question 4 How well prepared do you feel to work in a rural/remote area in the future? Pre = 2.6/5 (± 1.2), Post = 3.5/5 (± 0.7), P value = 0.003. Additional Post‐Placement Survey questions: Question 1: How would you rate your clinical experience on the trip? Question 2: How would you rate your cultural experience? Question 3: Has this trip changed your perception of Indigenous, rural, and remote health? Reflective Report quotes: ‘This was an invaluable opportunity to experience spectacular country but more importantly, remote health and to see first‐hand, the impact of cultural barriers on health care’. ‘I think now I have a much better understanding of the difficulties in Indigenous/remote health, feel I would be in a much better place to care for an Anangu Pitjantjatjara Yankunytjatjara Lands patients’. ‘I was also very privileged to meet one of the Elders of the Community who is active in the health service and who has a particular interest in mental health care and the uniqueness of Aboriginal mental health in particular.’
Webster et al. 2010 [29]	Pre‐ and post‐clinical placement questionnaire consisting of 10 items relating to diversity of experience, resources, support, access, feeling in control, feeling part of a team, feeling valued, maintaining work and family commitments, and financial costs associated with placement (5‐point likert scale). 4 open‐ended response questions related to their experiences during the placement and their suggestions for improving the students learning experience.	Students expressed a greater awareness and understanding of Aboriginal health issues as well as a substantial engagement with and support for the Aboriginal Community in which they were placed. 3 of 8 students wanted more preparation ‘I would have liked more exposure to Aboriginal health issues’, while another student commented that ‘I would like more tips on dealing with the Aboriginal Community’. Students also reported ‘I have learnt cultural sensitivity and respect for Aboriginal Australians’ and ‘I was able to learn about and value the roles of the Aboriginal Health worker’.
West et al. 2021 [20]	Survey (5‐point likert scale, 17 statements covering 4 domains of cultural awareness and capability). Domain 1: 3 statements; domain 2: 5 statements; domain 3: 5 statements; domain 4: 4 statements. Reflective journal.	Domain 1 Level of understanding of power relationships: median (IQR) Pre = 13/15 (11–14), Post = 14/15 (13–14), P value = <0.001, effect size = 0.51 (large effect). Domain 2 Level of understanding of the interrelationship between culture and self‐perceived health: median (IQR) Pre = 19/25 (17–22), Post = 22/25 (21–23), P value = <0.001, effect size = 0.59 (large effect). Domain 3 Level of understanding of the importance of culture in clinical practice and access to health care: median (IQR) Pre = 23/25 (21–24), Post = 24/25 (22–25), P value = <0.001, effect size = 0.39 (medium effect). Domain 4 Level of confidence with providing culturally safe care: median (IQR) Pre = 11/20 (9.75–15), Post = 17/20 (16–18), P value = <0.001, effect size = 0.61 (large effect).
Wright et al. 2014 [30]	Questionnaire (7‐point likert scale of 1–7 reported as mean value (±SD), 17 statements, investigated attitudes to rural practice) at the beginning and conclusion of the orientation, and on the final day of the placement. Focus group (semi‐structured interview).	Sample from Questionnaire: I am confident I could work in an Aboriginal Health Service: Pre = 2.9 /7 (± 1.6), Post = 3.6/7 (±1.5), P value = <0.001. I am not confident in my knowledge of Aboriginal health: Pre = 5.3/7 (± 1.4), Post = 4.3/7 (±1.6), P value = 0.013. Students who undertook a placement in Aboriginal health were more likely to understand Aboriginal health and the concepts of cultural safety and cultural security—‘. . . Because you can hear about it theoretically . . . But when you sort of see the doctors having to deal with it, and you see the patients that are walking in and walking out . . . It really brings it home’.

Abbreviations: ATIA, Attitudes Towards Indigenous Australians; IQR, interquartile range; MRS, Modern Racism Scale; RCS, Rural Clinical School; ±SD, ± one standard deviation.

### 3.3 Quality appraisal

Assessment using the Aboriginal and Torres Strait Islander QAT demonstrated that the reporting in the majority of studies to be incomplete (Supplementary File 3). Of note, negotiation of access to, and protection of, Aboriginal and Torres Strait Islander Peoples’ existing intellectual and cultural property, inclusion of Indigenous research paradigms, and Community control over the collection and management of research material were not clearly reported in the majority of included studies. More recently, published studies consistently performed better against the Aboriginal and Torres Strait Islander QAT reflecting growing recognition of the requirements for conducting ethical and respectful research with First Nations people.

In relation to the observational study appraisal checklist, all studies reported detailed information for study population, aims and outcomes, and study methods (Supplementary File 4). However, the least favorably ranked questions were those regarding whether the population studied were appropriate, if the results can be applied to the local situations, and if all likely effects could be seen in the timeframe. Qualitative studies also ranked low for questions regarding whether the author’s position was clearly stated, if the sampling strategy was clearly described and justified, and for the description and justification of procedures for data analysis/interpretation.

## 4 DISCUSSION

The aim of this systematic review was to evaluate whether participation in a clinical placement relating to health service delivery for Aboriginal and Torres Strait Islander people affects health professional students’ self‐perceived cultural capability. Fourteen studies were included that investigated pre‐placement to post‐placement self‐perceived cultural capability through a diverse range of study designs, clinical experiences, and health professions. Despite the different clinical experiences undertaken by students across a range of health professions, students consistently reported improvements in their self‐perceived cultural capability following participation in a clinical placement involving First Nations people and services.

The Framework states that ‘Good healthcare outcomes for Aboriginal and Torres Strait Islander Peoples require health professionals to be both clinically and culturally capable’ [10]. The findings of this review illustrate consistently across the studies that students self‐reported they became more aware and had greater understanding of the connection between culture, family, and Community and the importance of relationship building [19, 20, 21, 22, 24, 25, 27, 28, 29, 30]. In addition, students reported capacity to translate this understanding to support health care delivery and access, as well as recognise the barriers created by the discordance of mainstream health care models with First Nations health paradigms and ways of knowing, being, and doing [19, 20, 21, 22, 24, 25, 27, 28, 29, 30].

The intent of this review was to assess if clinical placement relating to health services for Aboriginal and Torres Strait Islander people affects health professional students’ self‐perceived cultural capability. However, this is a unidimensional assessment and represents a worldview of the students that is informed by social, economic, and political systems that perpetuate white privilege, and an education system that is informed by colonial constructs. These perceptions do not reflect valid measures of attitudinal change, cultural safety, or transformative unlearning where *‘unlearning is conceptualized within a transformative education paradigm, one whose primary orientation is discernment, a personal growth process involving the activities of receptivity, recognition and grieving’* [32].

As stated in the Strategy, cultural safety ‘is judged by the recipient of care, Aboriginal and Torres Strait Islander Peoples’ [7]. Although not the specific aim of this systematic review, there were little data reported in included studies in relation to the safety of the placement experience from the perspective of Aboriginal and Torres Strait Islander people, either as participants in delivering the placement, or those receiving care in the placement environment. Inclusion of these views is fundamental to determining if cultural capability training is effective to disrupt paternalistic approaches to health workforce training and clinical practice and should be a focus of future investigations. Only two studies included First Nations’ perceptions of student performance on the placement and the impact of the placement on service delivery in the published data set included in this systematic review. In their evaluation of a 2 month longitudinal placement, Bird et al. 2022 reported views of older Yolŋu as recipients of the service, families of older Yolŋu patients, as well as a manager of Community organisations who were immersed in the placement with students [25]. Students reported the placement improved their understanding of First Nations culture and delivery of culturally safe care as well as the importance of relationship building. This study, along with Askew et al. 2017 [21] and Bennet et al. 2013 [26], included placements longer than 4 weeks which may be important to allow relationship building by students with Community, and the reciprocity to support effective two‐way learning [25]. Consistent with this, West et al. 2021 has subsequently published an evaluation of a podiatry clinical service that supported the student placement experience, reported in this systematic review, from the perspective of First Nations patients. This subsequent study demonstrated patients of the co‐designed service valued their role as cultural educators of the students providing their care, and that the students attending were viewed as a positive aspect of the clinic: *“It’s breaking down the stereotypes … giving us the chance to talk to the students and teach them”* [33].

Clinical placements represent an integral part of training of health profession students. With increasing focus on development of culturally safe practitioners there is increasing demand for First Nations health placements. Western approaches to clinical education are typically training programs that are incorporated in an Australian healthcare model. This model promotes a singular approach to healthcare delivery that has been described as ‘a monocultural framework’ [34]. Indigenous led‐ and co‐designed approaches to delivery of culturally responsive health care training is essential to ensure self‐determination and empowerment of Aboriginal and Torres Strait Islander people, build reciprocity and respectful relationships between Communities and education providers, embed decolonising methodologies in clinical training programs and ensure cultural safety for First Nations health providers and the Communities they serve. First Nations‐led co‐design is a process that repositions power to First Nations Peoples, is founded in sustained engagement with First Nations Peoples and privileges and embeds First Nations knowledges, practices and priorities at every stage [35]. In this manner, First Nations‐led co‐design requires demonstrated commitment by non‐Indigenous people to engage in two‐way learning and lifelong development [36]. This protects against re‐articulation of colonising health care practices and unconscious preferencing of Western health care knowledge systems at the expense of First Nations epistemologies and ontologies [37, 38]. Most of the studies included in this systematic review had pre‐placement training and clinical placement experiences with attempted inclusion of elements of co‐design (Table 1). However, included studies noted difficulties with designing and implementing pre‐placement training and clinical placement experiences for entire cohorts of students including managing costs, limited resource capacity and mass accommodation requirements associated with their implementation [19, 21, 24, 26, 27, 29]. This reflects similar barriers to implementation of co‐designed Indigenous research, with the colonising approach and capitalistic axiology that has historically been reflective of Western academic institutions discordant with true commitment to co‐design [39, 40]. It is dismissive of the resources and time commitments that are essential to ensuring reciprocity, respect and inclusion of traditional practice, and the privileging and protection of First Nations knowledges to avoid misrepresentations through Western education and research practices, reporting and publishing [41, 42].

Although approximately 80% of Aboriginal and Torres Strait Islander people in Australia live in urban settings [43], only three [20, 21, 24] of the 14 included studies were set in urban locations. The clinical placement experience was also different for each study, as is expected with placements occurring in different First Nations. This positively exposes students to different Aboriginal and Torres Strait Islander Communities, the challenges, both health and social, they face and differing cultural practices and customs. Future clinical placements for health profession students need to be undertaken with and for First Nations organisations and Communities. Using First Nations‐led co‐design, these placement opportunities need to be developed from authentic and sustained relationships with First Nations health providers and Communities to meet their priorities and needs, with all stages of implementation and evaluation embedded in the co‐design process. The relationship between individual clinical placement components and self‐perceived cultural capability would be more conclusively established with evaluation that is inclusive of Indigenous research methodologies and privileges placement outcomes prioritised by First Nations people. Additional data collection methods including semi‐structured interviews or focus groups for non‐Indigenous students may also provide further insight into the effect of the clinical experience on health professional students’ cultural capability. Medium to long‐term follow‐up was performed by only three of the included studies [22, 25, 26], making it indeterminable as to whether the positive perceptions of increased cultural capability would remain with students for extended periods of time and beyond their tertiary education. Long‐term follow‐up would also facilitate evaluation of changes in workforce capabilities and distribution.

## 5 LIMITATIONS

This systematic review was designed to be comprehensive with a robust search of relevant databases, however it is possible that not all studies were identified particularly given the potential for studies to be within grey literature and the lack of structured search engines for these resources. Researchers in the field were not contacted for unpublished studies, authors were only contacted where information from included articles were missing or required clarification. There was no further information provided by some authors who were unable to be reached. Many studies examining self‐perceived cultural capability were excluded from this review as they lacked both pre‐ and post‐placement data. Nearly all studies used volunteer participants and are therefore subject to self‐select bias [44]. All studies displayed differing characteristics regarding cultural safety training, length of placement and type of placement experience, and the contribution that the individual components make to an increase in self‐perceived cultural capability is unknown.

The outcomes of this review are limited due to inconsistent reporting of Indigenous authorship, rights of access to, and protection of Aboriginal and Torres Strait Islander Peoples’ existing intellectual and cultural property and data sovereignty [16]. These are essential to ensuring the research was conducted and reported as a collaborative and culturally safe partnership between the researchers and the First Nations participants [45]. Decolonising research practice through promotion and facilitation of self‐determination, Community‐control, and Community ownership of research projects is central to ensuring research reflects Community health priorities and outcomes [46]. Additionally, researchers are encouraged to work with and for their local Aboriginal and Torres Strait Islander Communities and organisations to apply the principles of the Aboriginal and Torres Strait Islander QAT, including in the design, implementation, evaluation and reporting of their research [16]. Essential to interpreting the findings of this review is the need to consider outcomes of cultural capability or safety training evaluated from the perspectives of First Nations Peoples, using approaches determined appropriate by the local Aboriginal and Torres Strait Islander Community, before effectiveness or success of the program can be determined.

## 6 CONCLUSION

The results of this systematic review suggest that placements in health services or settings for Aboriginal and Torres Strait Islander Peoples that involve elements of co‐design are effective in increasing aspects of health professional students’ self‐perceived cultural capability. Further research is required to determine if these training experiences are culturally safe for First Nations people, and if they translate into sustained improvements in cultural capability of new health practitioners or result in increased cultural capability in provision of health services.

## AUTHOR CONTRIBUTIONS

**Kate Paisley**: conceptualisation; investigation; data curation; methodology; formal analysis; visualisation; writing—original draft; writing—review and editing. **Sean Sadler**: conceptualisation; investigation; data curation; methodology; formal analysis; visualisation; supervision; writing—original draft; writing—review and editing. **Matthew West**: conceptualisation; formal analysis; visualisation; writing—original draft; writing—review and editing. **James Gerrard**: conceptualisation; formal analysis; visualisation; writing—original draft; writing—review and editing. **Rhonda Wilson**: conceptualisation; formal analysis; visualisation; writing—original draft; writing—review and editing. **Angela Searle**: conceptualisation; investigation; data curation; methodology; formal analysis; visualisation; writing—original draft; writing—review and editing. **Vivienne Chuter**: conceptualisation; investigation; data curation; methodology; formal analysis; visualisation; supervision; writing—original draft; writing—review and editing.

## POSITIONALITY STATEMENT

MW (Wiradjuri) and RW (Wiradjuri) are proud First Nations Australians who bring Indigenous worldviews to this work. VC acknowledges her European and Māori ancestries, her Eurocentric dominant lived experience and her position as a learner in this space. Four authors are of European ancestry (KP, SS, JG, AS), and acknowledge Western knowledge systems, colonial lens, and the biases that accompany this worldview.

## CONFLICT OF INTEREST STATEMENT

The authors declare that they have no competing interests.

## ETHICS STATEMENT

Not applicable.

## Supporting information

Supporting Information S1

Supporting Information S2

Supporting Information S3

## Data Availability

Data supporting this study are included within the article and supporting information.
