# Supplementary material for: Determining health professional students’ self‐perceived cultural capability following participation in clinical placement with Aboriginal and Torres Strait Islander Peoples: A systematic review
Source: J Foot Ankle Res. 2024 Dec 9;17(4):e70017. doi: 10.1002/jfa2.70017 (PMC11628354; doi:10.1002/jfa2.70017)
Supplement: Supplementary file 1 — Supporting Information S1 [file JFA2-17-e70017-s001.docx]

**Supplementary File 1:** Search Terms

|  | SEARCH TERMS |
| --- | --- |
| 1. | Physician* or general practitioner* or surgeon* or allied health or doctor* or medical professional* or (health and (profession* or occupation* of personnel or care worker* or worker*)) or nurs* or midwife* or dentist* or dental or physio* or dietit* or dietic* or dietetic* or speech pathologist* or opthalomolog* or orthpotic* or audiolog* or occupational therap* or optom* or orthotic* or podiatr* or prosthetics or pharmac* or physio* or podiat* or psycholog* or radiograph* or social work* or speech patholog* or midwi* of medical or medic or medics or nutritionist* or diabet* educator* |
| 2. | Student* or undergraduat* or post graduat* or postgraduat* or PhD or higher degree research or master* or graduat* or diploma or prevocational or trainee |
| 3. | Placement* or clerkship* or training program* or intern* or experience* or practicum* or clinical practice* or fieldwork or workplace learning or work based learning or health education* or work integrated learning or immers* of cultur* or capability of competenc* |
| 4. | Aborigin* or indigenous or torres strait island* or ATSI or Koori* or Tiwi or first people* or nati* people or first nati* |
| 5. | 1 AND 2 AND 3 AND 4 |
|  |  |

| **Supplementary File 2:** Excluded Studies | | |
| --- | --- | --- |
| **Study** | **Name** | **Reason** |
| Abuzar et al 2009 | Development of a rural outplacement programme for dental undergraduates: Students’ perceptions. European Journal of Dental Education. November 2009; 13 (4): 233-239 | Wrong study design |
| Abuzar and Owen 2016 | A community engaged dental curriculum: A rural indigenous outplacement programme. Journal of Public Health Research. 26 April 2016; 5 (1): 27-31 | Wrong study design |
| Adams et al 2005 | Integrated support for Aboriginal tertiary students in health-related courses: the Pika Wiya Learning Centre. Australian health review: a publication of the Australian Hospital Association. Nov 2005; 29 (4): 482-488 | Wrong patient population |
| Alexander and Fraser 2005 | Professional career needs of GPs and registrars working in northwestern NSW. Australian Family Physician. Dec 2005; 34 Suppl 1 (): 7-9 | Wrong patient population |
| Amorin-Woods et al 2021 | Online or onsite? Comparison of the relative merit of delivery format of Aboriginal cultural-awareness-training to undergraduate chiropractic students. Journal for Multicultural Education 2021; 15(4): 374-394 | Wrong study design |
| Anderson et al 2009 | Indigenous medical workforce development: Current status and future directions. Medical Journal of Australia. 18 May 2009; 190 (10): 580-581 | Wrong outcomes |
| Angus et al 2016 | Aboriginal and Torres Strait Islander Public Health: Online and Integrated into Core Master of Public Health Subjects. Journal of Public Health Research. Apr 26 2016; 5 (1): 675 | Wrong outcomes |
| Anstice et al 2023 | Developing culturally safe education practices in optometry schools across Australia and Aotearoa New Zealand. Clinical & experimental optometry 2023 Vol. 106(2) Pages 110-118 | Wrong outcomes |
| Armitage and McMaster 2000 | Rural and remote mental health placements for nursing students. Australian Journal of Rural Health 2000; 8 (3): 175-179 Malden, Massachusetts Wiley-Blackwell 2000 | Wrong outcomes |
| Arnold et al 2008 | Incorporating cultural safety in nursing education. Nursing BC/Registered Nurses Association of British Columbia. Apr 2008; 40 (2): 14-17 | Wrong setting |
| Azer 2020 | Race and Culture in Teaching Cases. Academic Medicine. Feb 2020; 95 (2): 173-174 | Wrong study design |
| Balaratnasingam et al 2019 | Cultural intelligence throughout a lifetime. Australian and New Zealand Journal of Psychiatry. April 2019; 53 (Supplement 1):39 | Wrong outcomes |
| Barker et al 2022 | Co-creation of student-implemented allied health service in a First Nations remote community of East Arnhem Land, Australia. Australian Journal of Rural Health. Dec 2022; 30 (6): 782-794 | Wrong study design |
| Barraclough and Pit 2018 | Preparing the future workforce to address the health needs of small rural Australian towns through non-traditional allied health student placements. International Journal of Integrated Care (IJIC) 2018; 18: 1-2 Ubiquity Press 2018 | Wrong outcomes |
| Battye and McTaggart 2003 | Development of a model for sustainable delivery of outreach allied health services to remote north-west Queensland, Australia. Rural & Remote Health. Oct-Dec 2003; 3 (3): 194 | Wrong outcomes |
| Bazen et al 2007 | An innovation in Australian dental education: rural, remote and Indigenous pre-graduation placements. Rural and remote health. 2007; 7 (3): 703 | Wrong study design |
| Bazen et al 2007 | An Aboriginal and Torres Strait Islander oral health curriculum framework: Development experiences in Western Australia. Australian Dental Journal. June 2007; 52 (2): 86-92 | Wrong outcomes |
| Beecher et al 2010 | International students’ views about transferability in social work education and practice. International Social Work. 2010; 53 (2): 203-216 Sage Publications, Ltd. 2010 | Wrong setting |
| Begoray and Banister 2008 | Learning about Aboriginal contexts: the reading circle approach. J Nurs Educ. Jul 2008; 47 (7): 324-6 | Wrong outcomes |
| Bennett et al 2022 | Decolonization and trauma-informed truth-telling about indigenous Australia in a social work diversity course: A cultural safety approach. Journal of Ethnic and Cultural Diversity in Social Work: Innovation in Theory, Research & Practice. 2022 | Wrong study design |
| Bentley et al 2021 | Strengthening Indigenous eye care in Australia and New Zealand through a Leaders in Indigenous Optometry Education Network. Australian & New Zealand Journal of Public Health. 2021; 45 (2): 89-92 | Wrong outcomes |
| BergBlanc 2015 | Midwifery journey in Alice Springs. Australian Midwifery News. Winter 2015; 15 (2): 47-47 | Wrong study design |
| Biles et al 2021 | Aboriginal and Torres Strait Islander Subjects in a Graduate Diploma of Midwifery: A pilot study. Contemporary Nurse 01 Oct 2021; 57 (5):317-326 | Wrong setting |
| Biles 2018 | The development of Indigenous Australian cultural competence in nursing. Australian Nursing & Midwifery Journal. 2018; 26 (4): 40-41 | Wrong outcomes |
| Biles and Biles 2010 | Indigenous community participation: How does it relate to student centered learning and embrace primary health care philosophies? Contemporary Nurse: A Journal for the Australian Nursing Profession. 2010; 37 (1): 92-95 | Wrong outcomes |
| Biles 2018 | Strengthening relationships: We all need a place to call home. Emergency Medicine Australasia. 2018; 30 (6): 857-858 | Wrong study design |
| Birden and Wilson 2012 | Rural placements are effective for teaching medicine in Australia: evaluation of a cohort of students studying in rural placements. Rural and remote health. Oct 2012; 12 (4): 2167 | Wrong outcomes |
| Blake et al 2021 | Black, Indigenous, People of Colour, and International Students: Experiences and Resolutions Beyond COVID-19. American Journal of Public Health. 01 Mar 2021; 111 (3): 384-386 | Wrong outcomes |
| Bloomfield et al 2015 | Nursing students’ intentions to enter primary health care as a career option: Findings from a national survey. Collegian 2015; 22 (2): 161-167 | Wrong outcomes |
| Bolton and Andrews 2018 | ‘I learned more than from any lecture’ – Indigenous place and space for teaching Indigenous health to physiotherapy students. Physical Therapy Reviews. 2018; 23 (1): 35-39 | Wrong setting |
| Booth and Nelson 2013 | Sharing stories: using narratives to illustrate the role of critical reflections in practice with First Australians. Occupational Therapy International. 01 Sep 2013; 20 (3): 114-123 | Wrong patient population |
| Brideson and Kanowski 2004 | The struggle for systematic ‘adulthood’ for Aboriginal mental health in the mainstream: the Djirruwang Aboriginal and Torres Strait Islander Mental Health Program. Australian e-Journal for the Advancement of Mental Health. 2004; 3 (3): 1-9 | Wrong outcomes |
| Brighton et al 2011 | A strong commitment to mental health nursing. Australian Nursing Journal. 2011; 19 (4): 40-41 | Wrong outcomes |
| Brumpton et al 2021 | How can cultural safety, as determined by Aboriginal and Torres Strait Islander peoples, be assessed in GP trainees? Australian Journal of Primary Health 2021; 27(4) | Wrong patient population |
| Buhagiar et al 2023 | A pilot study to assess the impact of aboriginal and torres strait islander cultural humility webinars on australian medical school students. BMC medical education 2023 Vol. 23(1) Pages 626 | Wrong setting |
| Burley and Steers 2010 | A beneficial partnership: building the foundations for student interprofessional collaborative practice placements: a community health service and university partnership. Nursing Review (1326-0472) 2010; (): 16-17 | Wrong outcomes |
| Cairns et al 2022 | Developing a community rehabilitation and lifestyle service for a remote indigenous community. Disability and rehabilitation. Aug 2022; 44 (16): 4266-4274 | Wrong study design |
| Cairns et al 2021 | Developing a community rehabilitation and lifestyle service for a remote indigenous community. Disability and rehabilitation. 23 Mar 2021; (): 1-9 | Wrong outcomes |
| Cameron 2010 | Supporting Indigenous nursing students. Australian Nursing Journal. 2010; 18 (6): 39-39 | Wrong outcomes |
| Cameron et al 2015 | Remote supervision of medical training via videoconference in northern Australia: a qualitative study of the perspective of supervisors and trainees. BMJ Open. Mar 20 2015; 5 (3): e006444 | Wrong outcomes |
| Cedeno Betancourt and Keys 2021 | How important is it to be in a community that reflects your racial identity? Exploring the experiences of black indigenous and people of color (BIPOC) students in rural and underserved clinical settings. Journal of Investigative Medicine. 2021; 70 (): 294 | Wrong setting |
| Chang et al 2017 | Cultural competence education for health professionals from pre-graduate to licensure delivered using facebook: Twelve-month follow-up on a randomized control trial. Nurse Educ Today. Dec 2017; 59 (): 94-100 | Wrong setting |
| Chapman 2019 | Yarning Circles – their value in midwifery education in Western Australia. Women and Birth. September 2019;32(Supplement 1)():S45 | Full text not available |
| Chrisman 2007 | Extending cultural competence through systems change: academic, hospital, and community partnerships. J Transcult Nurs. Jan 2007; 18 (1 Suppl): 68S-76S; discussion 77S-85S | Wrong setting |
| Ciofalo et al 2022 | Indigenous community psychologies, decolonization, and radical imagination within ecologies of knowledges. American Journal of Community Psychology. 2022;11():2022 | Wrong outcomes |
| Clarke et al 2013 | Aboriginal mental health workshop: Make a difference? No we can. Australian and New Zealand Journal of Psychiatry. May 2013; 47 (): 24 | Wrong outcomes |
| Clarke 2012 | Understandings developed following ESTP to aboriginal medical service, MT Druitt. Australian and New Zealand Journal of Psychiatry. April 2012; 46 (): 8-9 | Wrong outcomes |
| Coleman et al 2021 | The experiences of Aboriginal and Torres Strait Islander Bachelor of Midwifery students: An integrative literature review. Women Birth. Feb 2021; 34 (1): 69-76 | Wrong study design |
| Coombe et al 2019 | Educating for Indigenous public health competence – how do we stack up in Australia? Australian and New Zealand Journal of Public Health. 01 Apr 2019; 43 (2): 143-148 | Wrong outcomes |
| Cord-Udy 2011 | Remote area indigenous psychiatry – Reflections on nine years as a visiting psychiatrist. Australian and New Zealand Journal of Psychiatry. May 2011; 45 (O1)(): A31 | Wrong outcomes |
| Cordes 2021 | Developing antiracist integrated health professionals. Families, Systems, & Health; 39 (2): 404-407 | Wrong study design |
| Courtenay 2005 | Seeking cultural competence: Aboriginal nursing students’ experiences with distance technology. 2005/01//;():9-10 | Duplicate |
| Courtenay et al 2005 | Appendix 4 – Seeking cultural competence: Aboriginal nursing students’ experiences with distance technology. 2005/01//;()36-39 | Wrong setting |
| Courtney et al 2000 | Australian Aboriginal trainee health service management program: a new initiative. Australian health review: a publication of the Australian Hospital Association. 2000; 23 (4): 97-107 | Wrong outcomes |
| Critchley et al 2007 | A required rural health module increases students’ interest in rural health careers. Rural & Remote Health. Apr-Jun 2007; 7 (2): 688 | Wrong study design |
| Crouch et al 2020 | Practitioner perceptions of the health of Australian First Nations’ Peoples: Preliminary findings. The Australian journal of rural health. 01 Aug 2020; 28 (4): 351-359 | Wrong outcomes |
| Crowshoe et al 2005 | Interactive drama: teaching aboriginal health medical education. Medical Education. May 2005; 39 (5): 521-2 | Wrong outcomes |
| Currie et al 2018 | Building Foundations for Indigenous Cultural Competence: An Institution’s Journey Toward “Closing the Gap”. Journal of Medical Imaging and Radiation Sciences. March 2018; 49 (1): 6-10 | Wrong outcomes |
| Darivemula et al 2021 | Dancing doctors: exploring the potential of cultural dance education to improve cultural competency and clinical communication. Public Health. June 2021; 195:22-23 | Wrong study design |
| Devlin et al 2022 | The bush to the beach: effect of rural placements on graduate practice location. Journal of Medical Radiation Sciences. May 2022; 69 (SUPPL 1):19 | Wrong study design |
| Doherty 1989 | Multiculturalism and medicine in Australia. Medical Journal of Australia. Nov 20 1989; 151 (10): 545-6 | Wrong outcomes |
| Doran and Wrigley 2022 | Cultural safety: Teachers engagement with an Indigenous pedagogical method in undergraduate nursing education. Contemporary Nurse. Feb 2022: 1-25 | Wrong patient population |
| Doran et al 2019 | Exploring cultural safety with Nurse Academics. Research findings suggest time to “step up”. Contemporary Nurse. 01 Apr 2019; 55 (2-3): 156-170 | Wrong patient population |
| Doran and Wrigley 2022 | ‘Teaching in Circle’ with student nurses contributes to experiential understanding of cultural safety. Contemporary Nurse. March 2022:1-26 | Wrong setting |
| Doran and Wrigley 2018 | Issues. Cultural Safety and Implications for Building Staff Capacity: Snapshot of findings from a study with nurse academics. Australian Nursing & Midwifery Journal. 2018; 25 (9): 22-22 | Wrong patient population |
| Duffy et al 2013 | Processes and outcomes for a successful engagement between a medical school and a remote Indigenous community in North Queensland, Australia. Rural and remote health. 2013; 13 (2): 2277 | Wrong outcomes |
| Durey 2010 | Reducing racism in Aboriginal health care in Australia: where does cultural education fit? Australian and New Zealand Journal of Public Health. Jul 2010; 34 Suppl 1 (): S87-92 | Wrong outcomes |
| Dyson et al 2014 | A decade of experience evolving visiting dental services in partnership with rural remote Aboriginal communities. Australian Dental Journal. June 2014; 59 (2): 187-192 | Wrong outcomes |
| Elliott 2013 | Student’s corner: providing culturally competent learning experiences for Aboriginal students: an undergraduate midwife’s perspective. Contemporary Nurse. Dec 2013; 46 (1): 139-142 | Wrong patient population |
| Eton 2012 | The value of experience in Aboriginal health. Australian Journal of Pharmacy. September 2019;93(1108):35 | Wrong study design |
| Ewen et al 2015 | When a patient’s ethnicity is declared, medical students’ decision-making processes are affected. Internal Medicine Journal. 2015; 45 (8): 805-812 | Wrong outcomes |
| Ewen and Hollinsworth 2016 | “Unwell while Aboriginal”: iatrogenesis in Australian medical education and clinical case management. Advances in Medical Education & Practice. 2016; 7 (): 311-5 | Wrong study design |
| Fatima et al 2018 | Positive placement experience and future rural practice intentions: Findings from a repeated cross-sectional study. Journal of Multidisciplinary Healthcare. 2018; 11(): 645-652 | Wrong study design |
| Fitzpatrick et al 2019 | Learning about Aboriginal health and wellbeing at the postgraduate level: novel application of the Growth and Empowerment Measure. Rural & Remote Health. 2019; 19 (2): 13-25 | Wrong setting |
| Fleming et al 2017 | Impact of continuing professional development intervention on midwifery academic’s awareness of cultural safety. Women and Birth. June 2017; 30 (3): 245-252 | Wrong patient population |
| Fleming et al 2019 | Cultural safety continuing professional development for midwifery academics: An integrative literature review. Women Birth. Aug 2019; 32 (4): 318-326 | Wrong study design |
| Fletcher 2019 | The National Rural Health Student Network: The future of rural health. Women and Birth. Sep 2019; 32 (Supplement 1) (): S2-S3 | Wrong study design |
| Forsyth et al 2019 | Students don’t know what they don’t know: Dental and Oral Health students’ perspectives on developing cultural competence regarding Indigenous Peoples. Journal of Dental Education. 01 Jun 2019; 83 (6): 679-686 | Wrong setting |
| Forsyth et al 2019 | Strengthening Indigenous cultural competence in dentistry and oral health education: Academic perspectives. European Journal of Dental Education: official journal of the Association for Dental Education in Europe. 01 Feb 2019; 23 (1): e37-e44 | Wrong patient population |
| Forsyth et al 2018 | Indigenous cultural competence: A dental faculty curriculum review. European Journal of Dental Education: official journal of the Association for Dental Education in Europe. 01 Aug 2018; 22 (3): e419-e426 | Wrong setting |
| Forsyth et al 2017 | Teaching cultural competence in dental education: a systematic review and exploration of implications for Indigenous populations in Australia. J Dent Educ. Aug 2017; 81 (8): 956-968 | Wrong study design |
| Forsyth et al 2020 | An Indigenous cultural competence model for dentistry education. Br Dent J. May 2020; 228 (9): 719-725 | Wrong setting |
| Foy and Tierney 2014 | Internal medicine in the bush: A clinical audit of a rural and remote outreach programme. Internal Medicine Journal. April 2014; 44 (4): 369-374 | Wrong outcomes |
| Francis-Cracknell et al 2019 | Indigenous health curriculum and health professional learners: a systematic review. Med Teach. May 2019; 41 (5): 525-531 | Wrong study design |
| Gadsden et al 2019 | Can a continuous quality improvement program create culturally safe emergency departments for Aboriginal people in Australia? A multiple baseline study. BMC Health Serv Res. Apr 11 2019; 19 (1): 222 | Wrong outcomes |
| Gair et al 2015 | Racism Unmasked: The experiences of Aboriginal and Torres Strait Islander students in social work field placements. Australian Social Work. 2015; 68 (1): 32-48 | Wrong patient population |
| Garvey et al 2009 | Indigenous Australian medical students’ perceptions of their medical school training. Medical Education. 2009; 43 (11): 1047-1055 | Wrong patient population |
| Garvey et al 2022 | Enhancing cultural capabilities amongst health professional students: a pilot study of interprofessional tag team simulation. Clinical Simulation in Nursing. 2022;62():83-91 | Wrong setting |
| Gilles et al 2008 | “If it wasn’t for OTDs, there would be no AMS”: overseas-trained doctors working in rural and remote Aboriginal health settings. Australian Health Review. Nov 2008; 32 (4): 655-63 | Wrong patient population |
| Gladman and Perkins 2013 | Training Australian general practitioners in rural public health: Impact, desirability and adaptability of hybrid problem-based learning. Health Education Journal. September 2013; 72(5): 522-529 | Wrong patient population |
| Gladman et al 2015 | Measuring organisational-level Aboriginal cultural climate to tailor cultural safety strategies. Rural and remote health. 01 Oct 2015; 15 (4): 3050 | Wrong patient population |
| Goold and Usher 2006 | Meeting the health needs of Indigenous people. How is nursing education meeting the challenge? Contemporary Nurse: A Journal for the Australian Nursing Profession. 2006; 22 (2): 288-295 | Wrong outcomes |
| Grant and Francis 2008 | Closed doors and culture wars: contemporary challenges for human services delivery in rural and regional Australia. Rural Social Work & Community Practice. 2008; 13 (1): 46-54 | Wrong patient population |
| Greenhill et al 2015 | Outcomes of Australian rural clinical schools: a decade of success building the rural medical workforce through the education and training continuum. Rural and remote health. 01 Jul 2015; 15 (3): 2291 | Wrong outcomes |
| Grootjans 2006 | Focus. Indigenous health/education: developing career pathways for Indigenous students. Australian Nursing Journal. 2006; 14 (2): 25-25 | Wrong outcomes |
| Gumley 2010 | A clinical experience: Kate and I. Australian Nursing Journal. 2010; 17 (7): 27-27 | Wrong study design |
| Gwynne and Lincoln 2017 | Developing the rural health workforce to improve Australian Aboriginal and Torres Strait Islander health outcomes: a systematic review. Aust Health Rev. May 2017; 41(2):234-238 | Wrong study design |
| Han et al 2003 | More rural and Aboriginal students for health-related university courses – are we making progress? Australian Health Review. 2003;26(2):73-7 | Wrong outcomes |
| Harman 2013 | University-based inter-professional health education initiative: Considering culture and pain in aboriginal people. Pain and Research Management. March-April 2013;18(2)():e3 | Wrong setting |
| Harms et al 2011 | Social work with Aboriginal clients: Perspectives on educational preparation and practice. Australian Social Work. 2011;64(2):156-168 | Wrong patient population |
| Hart et al 2015 | The “Strengthening Nursing Culture Project” – an exploratory evaluation study of nursing students’ placements within Aboriginal medical services. Contemp Nurse. Oct-Dec 2015;51(2-3):245-56 | Wrong study design |
| Harte et al 2011 | Tailoring registrar rural and remote general practice education for international medical graduates. Medical Education, Supplement. October 2011; 45():26 | Wrong outcomes |
| Harvey et al 2020 | Rural medical students’ self-reported perceptions of preparedness to practice in the Aboriginal and Torres Strait Islander Health Context. Australian Journal of Rural Health. 29(2):261-266 | Wrong setting |
| Hayes et al 1994 | Attitude change amongst nursing students towards Australian aborigines. International Journal of Nursing Studies. 1994; 31(1):67-76 | Wrong setting |
| Hays 2001 | Rural initiatives at the James Cook University School of Medicine: a vertically integrated regional/rural/remote medical education provider…Papers from the Australian college of rural and remote medicine inaugural scientific forum ‘steps along the pathway’, Brisbane, Queensland, 10 June 2001. Australian Journal of Rural Health. 2001;9():S2-5 | Wrong study design |
| Hays 2002 | One approach to improving indigenous health care through medical education. The Australian journal of rural health. Dec 2002;10(6):285-287 | Wrong outcomes |
| Hays 2013 | Utilisation of the healthcare system for authentic early experience placements. Rural and remote health. 2013;13(2):2328 | Wrong outcomes |
| Henry et al 2009 | Why do medical graduates choose rural careers? Rural Remote Health. Jan-Mar 2009;9(1):1083 | Wrong outcomes |
| Herceg and Renouf 2016 | Teaching near and far – Broome, Western Australia. Cureus. Nov 09 2016;8(11):e870 | Wrong study design |
| Herzog et al 2021 | The KAIROS Blanket Exercise: engaging Indigenous ways of knowing to foster critical consciousness in medical education. Medical Teacher. 43(12):1437-1443 | Wrong setting |
| Hill et al 2017 | Development of student clinics in Indigenous contexts. Journal of Clinical Practice in Speech-Language Pathology. 2017;19(1):40-45 | Wrong study design |
| Hills et al 2019 | Core indicators of quality in practice education placements in allied health and social care professions: a scoping review protocol. JBI Database System Rev Implement Rep. Jun 2019;17(6):1060-1070 | Wrong study design |
| Hinton and Chirgwin 2010 | Nursing education: reducing reality shock for graduate Indigenous nurses – it’s all about time. Australian Journal of Advanced Nursing. 2010;28(1):60-66 | Wrong patient population |
| Hoad and Hayward 2012 | Student Aboriginal health worker smoking: findings from a training college in Western Australia. Australia and New Zealand Journal of Public Health. 2012;36(3):296-297 | Wrong outcomes |
| Hofer et al 2014 | Prevocational exposure to public health in the Kimberley: a pathway to rural, remote and public health practice. The Australian journal of rural health. 01 Apr 2014;22(2):75-79 | Wrong outcomes |
| Hoffart et al 2013 | Intraprofessional practice education using a community partnership model. Journal of Nursing Education. 2013;52(2):104-107 | Wrong patient population |
| Holst 2020 | Increasing rural recruitment and retention through rural exposure during undergraduate training: an integrative review. Int J Environ Res Public Health. Sep 3 2020;17(17) | Wrong study design |
| Howe-Murphy et al 1989 | Effecting change in multicultural health promotion: a systems approach. Journal of Allied Health. 1989;18(3):291-305 | Wrong outcomes |
| Humphreys et al 2000 | Policy issues. Roles and activities of the Commonwealth Government University Departments of Rural Health. Australian Journal of Rural Health. 2000;8(2):120-133 | Wrong study design |
| Hunt et al 2015 | Nursing student’s perspective of the health and healthcare issues of Australian Indigenous people. Nurse Education Today. 01 Mar 2015;35(3):461-467 | Wrong setting |
| Immerman 2012 | You and me. Medical Journal of Australia. 20 Feb 2012; 196(3):204-205 | Wrong study design |
| Isaacs et al 2016 | Cultural desire need not improve with cultural knowledge: A cross-sectional study of student nurses. Nurse Education in Practice. 01 Jul 2016;19():91-96 | Wrong setting |
| Jackson et al 2013 | Amazingly resilient Indigenous people! Using transformative learning to facilitate positive student engagement with sensitive material. Contemporary Nurse. Dec 2013;46(1):105-112 | Wrong setting |
| Jacob et al 2016 | Exploration of nursing degree students’ content expectations of a dedicated Indigenous health unit. Collegian. 2016;23(3):313-319 | Wrong outcomes |
| Jeffery et al 2014 | The value of best-practice guidelines for OSCEs in a postgraduate program in an Australian remote area setting…Objective structured clinical examinations. Rural and Remote Health. 2014;14(3):1-9 | Wrong setting |
| John et al 2014 | Development and evaluation of successful multicultural training. Australian and New Zealand Journal of Psychiatry. May 2014;48():60 | Wrong patient population |
| Johnson and Withers 2018 | Cultural competence in the emergency department: Clinicians as cultural learners. Emergency Medicine Australasia. 2018;30(6):854-856 | Wrong study design |
| Kalfoglou and Sung 2002 | What inspires clinical research trainees and keeps them on the path? J Investig Med. Nov 2002;50(6):408-11 | Wrong outcomes |
| Kavaler 1992 | The experience of an American medical student with an aboriginal community in central Australia. New York State Journal of Medicine. 1992;92(10):431-435 | Wrong setting |
| Kelly and Henschke 2019 | The experiences of Australian indigenous nursing students: A phenomenological study. Nurse Education in Practice. 01 Nov 2019;41():102642 | Wrong patient population |
| Kelly et al 2014 | ‘She knows how we feel’: Australian Aboriginal and Torres Strait Islander childbearing women’s experience of Continuity of Care with an Australian Aboriginal and Torres Strait Islander midwifery student. Women and Birth. Sep 2014;27(3):157-162 | Wrong patient population |
| Kelly et al 2016 | Improving cultural and clinical competency and safety of renal nurse education. Renal Society of Australasia Journal. 2016;12(3):106-112 | Wrong setting |
| Kelly et al 2013 | Exploring the experiences of Indigenous women and Indigenous students who participated in a ‘follow through’ journey within a Bachelor of Midwifery Program. Women & Birth. 2013;26():S10-S10 | Wrong patient population |
| Kelly and Lazenby 2019 | Developing and validating learning domains, competencies, and evaluation items for global health clinical immersion practicums for graduate-level nursing programs. J Adv Nurs. Jan 2019;75(1):234-252 | Wrong study design |
| Kent-Wilkinson et al 2010 | International nursing student exchange: Rural and remote clinical experiences in Australia. Journal of Agromedicine. January 2010;15(1):58-65 | Wrong study design |
| Kerr et al 2020 | A scoping review of health professional curricula: Implications for developing integration in pharmacy. Res Social Adm Pharm. Jan 2020;16(1):1-16 | Wrong study design |
| Kerrigan et al 2024 | Evaluating the impact of 'Ask the Specialist Plus': a training program for improving cultural safety and communication in hospital-based healthcare. BMC health services research 2024 Vol. 24(1) Pages 119 | Wrong setting |
| Kickett et al 2014 | A model for large-scale, interprofessional, compulsory cross-cultural education with an indigenous focus. Journal of Allied Health. Spring 2014;43(1):38-44 | Wrong setting |
| Kildea et al 2018 | Birthing on Country (in our community): a case study of engaging stakeholders and developing a best-practice Indigenous maternity service in an urban setting. Australian health review: a publication of the Australian Hospital Association. 01 Apr 2018;42(2):230-238 | Wrong study design |
| King et al 2012 | The experiences of remote and rural aboriginal health workers and registered nurses who undertook a postgraduate diabetes course to improve the health of Indigenous Australians. Contemporary Nurse. 2012;42(1):107-117 | Wrong patient population |
| Kirby et al 2018 | Growing health partnerships in rural and remote communities: what drives the joint efforts of primary schools and universities in maintain service learning partnerships? Primary Health Care Research & Development. 01 Sep 2018;19(5):503-517 | Wrong outcomes |
| Kirkby 2018 | Midwifery students’ perception of their ability to provide culturally safe maternity care for Australian Aboriginal women. Australian Nursing & Midwifery Journal. 2018;26(4):38-38 | Full text not available |
| Kirkham et al 2005 | Narratives of social justice: Learning in innovative clinical settings. International Journal of Nursing Education Scholarship. 02 Dec 2005;2(1):i-14 | Wrong setting |
| Kristof et al 2017 | Preparing indigenous students to enter health science professions. Journal of Medical Radiation Sciences. March 2017;64 (Supplement 1):107 | Wrong study design |
| Kruger and Tennant 2010 | Short-stay rural and remote placements in dental education, an effective model for rural exposure: a review of eight-year experience in Western Australia. Australian Journal of Rural Health. Aug 2010;18(4):148-52 | Wrong outcomes |
| Kuipers et al 2014 | Aboriginal and Torres Strait Islander health practitioners in rural areas: credentialing, context and capacity building. Rural and remote health. 2014;14(4):2897 | Wrong outcomes |
| Kurtz et al 2018 | Health sciences cultural safety education in Australia, Canada, New Zealand, and the United States: a literature review. Int J Med Educ. Oct 25 2018;9();271-285 | Wrong study design |
| Laddipeerla et al 2015 | Reflections on Aboriginal perinatal mental health, mothers, babies, families and community: A South Australian trainee’s experience. Australasian psychiatry: bulletin of Royal Australian and New Zealand College of Psychiatrists. 01 Dec 2015;23(6):693-695 | Wrong outcomes |
| Lalloo et al 2013 | Dental students’ reflections on clinical placement in a rural and indigenous community in Australia. Journal of Dental Education. Sep 2013;77(9):1193-1201 | Wrong study design |
| Lalloo et al 2013 | Dental care provision by students on a remote rural clinical placement. Australian and New Zealand Journal of Public Health. Feb 2013;37(1):47-51 | Wrong outcomes |
| Lawson et al 2007 | Training Indigenous doctors for Australia: shooting for goal. Medical Journal of Australia. May 21 2007;186(10):547-50 | Wrong patient population |
| Leibbrandt et al 2005 | National comparative curriculum evaluation of baccalaureate nursing degrees: a framework for the practice based professions. Nurse Education Today. 2005;25(6):418-429 | Wrong outcomes |
| Leininger 1984 | Transcultural nursing: an overview. Nursing Outlook. Mar-Apr 1984;32(2):72-3 | Wrong study design |
| Lennon et al 2019 | Attracting junior doctors to rural centres: A national study of work-life conditions and satisfaction. The Australian journal of rural health. 01 Dec 2019;27(6):482-488 | Wrong patient population |
| Lenthall et al 2022 | Reflections on an evaluation of the ‘Remote Health Experience” – an interprofessional learning, cultural immersion program. Rural & Remote Health. 2022; 22(4):1-9 | Wrong study design |
| Li et al 2017 | The Aboriginal Population Health Training Initiative: a NSW health program established to strengthen the Aboriginal public health workforce. Public Health Research & Practice. 2017;27(4) | Wrong patient population |
| Liaw et al 2005 | A compulsory experiential and interprofessional rural health subject for undergraduate students. Rural & Remote Health. Oct-Dec 2005;5(4):460 | Wrong outcomes |
| Lucas et al 2021 | Pharmacy students’ learnings and reflections to inform the development of the ‘Working Respectfully with Aboriginal Peoples’ (WRAP) Toolkit. Reflective Practice. 2021;22(1):86-100 | Wrong patient population |
| Lucas et al 2021 | Clinical partners’ reflections on Indigenous curricula in health education and the development of a pre-clinical placement student toolkit. Reflective Practice. 2021;22(2):263-277 | Wrong patient population |
| Main et al 2000 | Reconciling pedagogy and health sciences to promote Indigenous health. Australian and New Zealand Journal of Public Health. 2000;24(2):211-213 | Wrong study design |
| Mak and Plant 2005 | Reducing unmet needs: A prevocational medical training program in public health medicine and primary health care in remote Australia. Australian Journal of Rural Health. June 2005;13(3):183-190 | Wrong patient population |
| Mak et al 2006 | “I have learnt…A different way of looking at people’s health”: An evaluation of a prevocational medical training program in public health medicine and primary health care in remote Australia. Medical Teacher. Sep 2006;28(6):e149-e155 | Wrong study design |
| Mak et al 2013 | Prevocational exposure to public health in the Kimberley. Internal Medicine Journal. May 2013;43():46 | Wrong outcomes |
| Mak and Plant 2001 | John Flynn Scholarship students: case studies of useful contributions to remote health care. Australian Journal of Rural Health. 2001;9(5):246-250 | Wrong study design |
| Mangoyana et al 2022 | Positive oral health outcomes: A partnership model improves care in a rural Indigenous community. Australian Journal of Rural Health. Dec 2022;30():30 | Wrong patient population |
| March et al 2023 | Positive impacts of oral health services provision by a student-led primary care clinic to an Australian rural indigenous community. Australian dental journal 2023 Vol. 68(3) Pages 151-159 | Wrong outcomes |
| Mazel and Ewen 2015 | Innovation in Indigenous Health and Medical Education: The Leaders in Indigenous Medical Education (LIME) Network as a Community of Practice. Teaching and Learning in Medicine. 2015;27(3):314-328 | Wrong outcomes |
| McCalman et al 2017 | Organisational systems’ approaches to improving cultural competence in healthcare: a systematic scoping review of the literature. International Journal for Equity in Health. 2017;16(1) | Wrong study design |
| McChlery 2019 | Challenges in the implementation of a new course on aboriginal history, health, wellness and culture in a graduate diploma of midwifery program. Women and Birth. Sep 2019;32(Supplement 1):S39 | Wrong outcomes |
| McChlery 2019 | Challenges in the implementation of a new course on aboriginal history, health, wellness and culture in a graduate diploma of midwifery program. Women and Birth. Sep 2019;32(Supplement 1):S39 | Duplicate |
| McCleland 2011 | Culturally safe nursing research: Exploring the use of an indigenous research methodology from an indigenous researcher’s perspective. Journal of Transcultural Nursing. Oct 2011;22(4):362-367 | Wrong outcomes |
| McDonald et al 2018 | Transformative effects of Aboriginal health placements for medica, nursing, and allied health students: A systematic review. Nurs Health Sci. Jun 2018; 20(2):154-164 | Wrong study design |
| McDonnel Smedts et al 2007 | Clinical training in the top end: impact of the Northern Territory Clinical School, Australia, on the Territory’s health workforce. Rural and remote health. 2007;7(2);723 | Wrong outcomes |
| McGrail and O’Sullivan 2021 | Increasing doctors working in specific rural regions through selection from and training in the same region: national evidence from Australia. Human resources for health. Oct 2021;19(1):132 | Wrong patient population |
| McNaught et al 2023 | Exploring medical students’ rural intention on course entry. Australian and International Journal of Rural Education 2023 Vol. 33 Issue 3 Pages 47-61 | Wrong outcomes |
| McRae et al 2023 | Culturally supported health promotion to See, Treat, Prevent (SToP) skin infections in Aboriginal children living in the Kimberley region of Western Australia: a qualitative analysis. The Lancet Regional Health. Western Pacific Vol. 35 Pages | Wrong outcomes |
| Meissner 2010 | Exploring indigenous health using the clinical reasoning cycle: a student paper. Contemporary nurse: a journal for the Australian nursing profession. 2010;37(1):82-89 | Wrong outcomes |
| Merrifield 2017 | First student nurses from national care experience pilot due to graduate. Nursing times. 2017;113(3):1-3 | Wrong setting |
| Middleton et al 2017 | Incorporating the Nursing and Midwifery Aboriginal and Torres Strait Islander health curriculum framework into a Bn Program. Australian Nursing & Midwifery Journal. 01 Apr 2017;24(9):44 | Wrong study design |
| Miller et al 2018 | Emergency Medicine Student End-of-Rotation Examinations. Where are we now? West J Emerg Med. Jan 2018;19(1):134-136 | Wrong setting |
| Mills et al 2021 | Examining the transformative potential of emotion in education: A new measure of nursing and midwifery students’ emotional learning in first peoples’ cultural safety. Nurse Education Today. 01 May 2021;100():104854 | Wrong setting |
| Mills et al 2018 | Experiences and outcomes of health professional students undertaking education on Indigenous health: a systematic integrative literature review. Nurse Educ Today. Oct 2018;69():149-158 | Wrong study design |
| Mills et al 2022 | Evaluation of a First Peoples-led, emotion-based pedagogical intervention to promote cultural safety in undergraduate non-Indigenous health professional students. Nurse Education Today. 109():105219 | Wrong setting |
| Mills et al 2023 | Stories from the river: Thematic analysis of non-Indigenous health students’ free-text survey responses about Australian cultural safety education. AlterNative 2023 Vol. 19 Issue 1 Pages 32-41 | Wrong setting |
| Milne et al 2016 | Development of the awareness of cultural safety scale: a pilot study with midwifery and nursing academics. Nurse Education Today. 01 Sep 2016;44():20-25 | Wrong patient population |
| Mitchell et al 2022 | How immersion in remote Aboriginal communities influences medical students’ career intentions. Australian Journal of Primary Health. 2022;28(5): 380-386 | Wrong setting |
| Moffitt and Durnford 2021 | Undergraduate nursing students’ perceptions about creating culturally safe classrooms: Living the epistemology, ontology, and pedagogy. Nurse Education Today. Oct 2022;105():105029 | Wrong setting |
| Morgan 2006 | Orientation for general practice in remote Aboriginal communities: a program for registrars in the Northern Territory. Australian Journal of Rural Health. Oct 2006;14(5):202-208 | Wrong patient population |
| Morgan et al 2009 | From the bush to the big smoke – development of a hybrid urban community based medical education program in the Northern Territory, Australia. Rural and remote health. 2009;9(3):1175 | Wrong outcomes |
| Murray et al 2012 | Medical schools as agents of change: Socially accountable medical education. Medical Journal of Australia. June 2012;196(10):1-5 | Wrong study design |
| Murray and Wronski 2006 | When the tide goes out: Health workforce in rural, remote and indigenous communities. Medical Journal of Australia. 03 Jul 2006;185(1):37-38 | Wrong outcomes |
| Murry et al 2021 | Indigenous mentorship in the health sciences: actions and approaches of mentors. Teaching and learning in medicine. May 2021;():1-11 | Wrong setting |
| Nagel et al 2009 | Two way approaches to indigenous mental health approaches: brief training in brief interventions. Australian e-Journal for the Advancement of Mental Health. 2009;8(2):7p-7p | Wrong patient population |
| Nash et al 2006 | The Yapunyah project: embedding Aboriginal and Torres Strait Islander perspectives in the nursing curriculum. Contemporary nurse: a journal for the Australian nursing profession. Sep 2006;22(2):296-316 | Wrong setting |
| Newbury et al 2005 | Development of ‘rural week’ for medical students: impact and quality report. Rural and remote health. 2005;5(3):432 | Wrong study design |
| Newman 1993 | Nurses…bridging the gap: Australian aboriginals and primary health care. Journal of the Royal Society of Health. Apr 1993;113(2):87-90 | Wrong study design |
| Newton 2021 | Creating cultural safety as an Aboriginal teacher in a class of non-Aboriginal university students. Australian Social Work. 2021;74(1):4-12 | Wrong patient population |
| Nielsen 2011 | Safeguard or mollycoddle? Medical student placements in Aboriginal communities. Comment. The Medical journal of Australia. 18 Jul 2011;195(2):103 | Wrong study design |
| Nielsen 2011 | Safeguard or mollycoddle? Medical student placements in aboriginal communities. Medical Journal of Australia. July 2011;195(2):102-103 | Wrong study design |
| Norbye et al 2018 | Under the same sky: connecting students and cultures through circumpolar nursing education. Healthcare. May 21 2018;6(2):21 | Wrong setting |
| O’Mara 2009 | Close the gap: Australian indigenous doctors’ association. Medical Journal of Australia. 18 May 2009;190(10):607 | Wrong study design |
| O’Sullivan et al 2018 | A review of characteristics and outcomes of Australia’s undergraduate medical education rural immersion programs. Hum Resour Health. Jan 31 2018;16(1):8 | Wrong study design |
| Omeri and Ahern 1999 | Utilising culturally congruent strategies to enhance recruitment and retention of Australian indigenous nursing students. Journal of transcultural nursing. Apr 1999;10(2):150-155 | Wrong outcomes |
| Omeri et al 2003 | Meeting the challenges of cultural diversity in the academic setting. Nurse Education in Practice. Mar 2003;3(1):5-22 | Wrong study design |
| Ong 2013 | Aboriginal nursing & midwifery cadetships. Australian nursing & midwifery journal. 2013;21(6):36-37 | Wrong patient population |
| Ostini et al 2021 | Building a sustainable rural physician workforce. Medical Journal of Australia; 215 Suppl 1():S5-S33 | Wrong patient population |
| Page and Hamilton 2015 | Pharmacy students perceptions of a non-traditional rural placement: A pilot programme. Pharmacy Education. 01 Jan 2015;15(1):275-280 | Wrong study design |
| Palmer 1997 | Engendering cultural sensitivity in nursing students. The Australian and New Zealand journal of mental health nursing. Jun 1997;6(2):66-72 | Wrong setting |
| Passi 2018 | Looking forward, looking back: An Indigenous trainee perspective. Emergency Medicine Australasia. 2018;30(6):862-863 | Wrong study design |
| Patel et al 2011 | Safeguard or mollycoddle? An exploratory study describing potentially harmful incidents during medical student placements in aboriginal communities in central Australia. Medical Journal of Australia. 2011;194(10):497-500 | Wrong outcomes |
| Patel et al 2011 | Safeguard or mollycoddle? Medical student placements in aboriginal communities. Medical Journal of Australia. July 2011;195(2):104 | Duplicate |
| Patel et al 2023 | The Kimberley Dental Team: a volunteer-based model of care serving remote Aboriginal communities. Rural and remote health 2023 Vol. 23(3) | Wrong outcomes |
| Pederson and Barlow 2008 | Theory to social action: a university-based strategy targeting prejudice against Aboriginal Australians. Australian Psychologist. 2008;43(3):148-159 | Wrong setting |
| Penn 2014 | Recognising cultural safety issues for indigenous students in a baccalaureate nursing programme: two unique programmes. Whitireia Nursing & Health Journal. 2014;(21):29-33 | Wrong outcomes |
| Peterson et al 2010 | Marketing of rural and remote pharmacy practice via the digital medium. Journal of Clinical Pharmacy and Therapeutics. August 2010;35(4):409-414 | Wrong setting |
| Petric et al 2022 | “Bridging two worlds?”: Towards cultural safety within Schools of Nursing in Australian Universities. Nurse Education Today. March 2022;110():105176 | Wrong setting |
| Philips et al 1978 | Migration of allied health care personnel in and out of an underserved area: a question of roots. Journal of Allied Health. 1978;7(4):288-93 | Wrong study design |
| Pierce et al 2016 | Mental health academics in rural and remote Australia. Rural & Remote Health. 2016;13(3):1-11 | Wrong outcomes |
| Pierce et al 2016 | Mental health academics in rural and remote Australia. Rural & Remote Health. Jul-Sep 2016;16(3):3793 | Duplicate |
| Piercey and Robinson 2017 | Indigenous Nursing Workforce, to achieve equality in healthcare services. Australian Nursing & Midwifery Journal. 01 Apr 2017; 24(9):39 | Wrong outcomes |
| Piercey and Robinson 2017 | Indigenous Health Focus. Indigenous nursing workforce, to achieve equality in healthcare services. Australian Nursing & Midwifery Journal. 2017;24(9);39-39 | Wrong study design |
| Pitama et al 2018 | Implementation and impact of indigenous health curricula: a systematic review. Med Educ. Jun 22 2018 | Wrong study design |
| Pitama et al 2018 | Implementation and impact of indigenous health curricula: a systematic review. Medical Education. Jun 22 2018 | Duplicate |
| Pool and Macharouthu 2022 | Preparing the Future Workforce: improving medical student understanding of older persons’ mental health in regional, rural, and remote Australia. Australian and New Zealand Journal of Psychiatry. May 2022;56(SUPPL 1)():214 | Wrong study design |
| Powell 2018 | The power of diversity. Nature. 07 Jun 2018;558(7708):19-22 | Wrong study design |
| Power et al 2016 | REM: A collaborative framework for building indigenous cultural competence. Journal of transcultural nursing. 01 Sep 2016;27(5)239-446 | Wrong setting |
| Power et al 2013 | Editorial: creating connections: enhancing indigenous education through collaboration. Contemporary Nurse: A journal for the Australian Nursing Profession. 2013;46(1):95-96 | Wrong study design |
| Power et al 2016 | REM. Journal of Transcultural Nursing. 2016;27(5):439-446 | Duplicate |
| Power et al 2021 | Cultural safety and Indigenous authority in nursing and midwifery education and practice. Contemporary Nurse. Oct 2021;57(5)():303-307 | Wrong study design |
| Prentice et al 2023 | 'Being in a place where it matters': GPs who do and do not work in Aboriginal Health Services. Australian Journal of Primary Health Vol. 30 Issue 1 | Wrong patient population |
| Prout et al 2014 | ‘I could never have learned this in a lecture’: transformative learning in rural health education. Advances in health sciences education: theory and practice. 01 May 2014; 19(2):147-159 | Wrong setting |
| Pugh et al 2018 | Adaption and pretesting of the College Persistence Questionnaire V3 (Short Form) for measuring intention to persist among Aboriginal Diploma of Nursing Students. Nurse Education Today. 01 Feb 2018; 61():162-168 | Wrong patient population |
| Purea et al 2022 | Exploring the learning environment afforded by an Aboriginal Community Controlled Health service in a rural longitudinal integrated clerkship. Education for primary care: an official publication of the Association of Course Organizers, National Association of GP Tutors, World Organisation of Family Doctors. Mar 2022;():1-7 | Wrong study design |
| Rae et al 2016 | Cultural experiences of student and new-graduate dietitians in the Gomeroi gaaynggal ArtsHealth program: a quality assurance project. Health promotion journal of Australia. 20 Apr 2016;27(2):162-166 | Wrong study design |
| Ramjan et al 2016 | Predictors of negative attitudes toward Indigenous Australians and a unit of study among undergraduate nursing students: A mixed-methods study. Nurse Education in Practice. 01 Mar 2016;17():200-207 | Wrong setting |
| Ramsay and Kermode 1997 | Nurses facilitating reconciliation through education… a unit of study, health and Australian indigenous peoples. Australian Journal of Advanced Nursing. 1997;15(1):32-39 | Wrong setting |
| Ranzijn et al 2008 | Towards cultural competence: Australian Indigenous content in undergraduate psychology. Australian Psychologist. 2008;43(2):132-139 | Wrong setting |
| Raymond 2008 | Developing a new bachelor of nursing course responsive to Australia’s culturally diverse community. Contemporary Nurse: A journal for the Australian nursing profession. 2008;28(1-2):17-22 | Wrong setting |
| Rigby et al 2010 | Closing the Gap: cultural safety in Indigenous health education. Contemporary nurse: a journal for the Australian nursing profession. 2010;37(1):21-30 | Wrong outcomes |
| Rind 2017 | Feature. Beating the odds: how indigenous nurses and midwives are closing the health gap. Australian Nursing & Midwifery Journal. 2017;24(11):20-25 | Wrong study design |
| Robinson et al 2016 | ‘A foot in each world, a heart in none’ – whose responsibility is cultural competence? Australian & New Zealand Journal of Public Health. 2016;40(4):303-303 | Wrong study design |
| Sargeant et al 2016 | Enhancing cultural awareness education for undergraduate medical students: Initial findings from a unique cultural immersion activity. Australasian Medical Journal. 2016;9(7):224-230 | Wrong setting |
| Sarovich et al 2022 | Different meanings…what we want in our lives…a qualitative exploration of the experience of Aboriginal and/or Torres Strait Islander peoples in a co-designed community rehabilitation service. Disability and Rehabilitation. Dec 2022;():1-8 | Wrong patient population |
| Schulz et al 2018 | Evaluation of strategies designed to enhance student engagement and success of indigenous midwifery students in an Away-from-base- bachelor of midwifery program in Australia: a qualitative research study. Nurse Education Today. 01 Apr 2018;63():59-63 | Wrong patient population |
| Sen Gupta 2006 | Rural internship for final-year medical students. Medical Journal of Australia. 03 Jul 2006;185(1):54-55 | Wrong outcomes |
| Sen Gupta et al 2008 | Rural internships for final year students: clinical experience, education, and workforce. Rural and remote health. 2008;8(1):827 | Wrong outcomes |
| Sharmil et al 2021 | Participatory Action Research-Dadirri-Ganma, using yarning: methodology co-design with Aboriginal community members. International Journal for Equity in Health. 20(1):160 | Wrong patient population |
| Shattell 2007 | Engaging students and faculty with diverse first-person experiences: use of an interpretive research group. J Nurs Educ. Dec 2007;46(12):572-5 | Wrong setting |
| Sinnott and Wittmann 2001 | An introduction to indigenous health and culture: the first tier of the three tiered plan. Aust J Rural Health. Jun 2001;9(3):116-20 | Wrong patient population |
| Silversten et al 2017 | Indigenous health focus. Challenges to indigenous health curriculum design – bringing the Aboriginal and Torres Strait Islander health curriculum framework to life. Australian Nursing & Midwifery Journal. 2017;24(9):41-41 | Wrong setting |
| Skinner et al 2021 | A graduate oral health therapist program to support dental service delivery and oral health promotion in Aboriginal communities in New South Wales, Australia. Rural and remote health. 01 Jan 2021;21(1):5789 | Wrong outcomes |
| Slayter et al 2016 | Barriers and enablers to retention of Aboriginal Diploma of Nursing students in Western Australia: an exploratory descriptive study. Nurse Education Today. -1 Jul 2016;42():17-22 | Wrong patient population |
| Smith et al 2015 | Using cultural immersion as the platform for teaching Aboriginal and Torres Strait Islander health in an undergraduate medical curriculum. Rural and remote health. 01 Jul 2015;15(3):3144 | Wrong setting |
| Smith et al 2018 | Health professional students’ rural placement satisfaction and rural practice intentions: A national cross-sectional survey. The Australian journal of rural health. 01 Feb 2018;26(1):26-32 | Wrong study design |
| Spencer 2011 | Thoughts and feelings. Nursing Standard. 2011;26(11):64-64 | Wrong study design |
| Spiers and Harris 2015 | Challenges to student transition in allied health undergraduate education in the Australian rural and remote context: a synthesis of barriers and enablers. Rural & remote health. 2015;15(2):1-17 | Wrong study design |
| Stansfield and Browne 2013 | The relevance of indigenous knowledge for nursing curriculum. Int J Nurs Educ Scholarsh. Jun 25 2013;10 | Wrong setting |
| Stuart and Sengstock 2017 | Focus Indigenous Health. How can schools of nursing and midwifery prepare their graduates to encourage medication adherence for Australian aboriginal and Torres Strait Islander clients? Australian Nursing & Midwifery Journal. 2017;24(9):42-42 | Wrong outcomes |
| Stuhlmiller and Tolchard 2015 | Developing a student-led health and wellbeing clinic in an underserved community: collaborative learning, health outcomes and cost saving. BMC Nursing. 2015;14():32 | Wrong outcomes |
| Svarc et al 2018 | Exploring the impact of Aboriginal health placement experiences on the preparation of dietetic graduates for practice with Aboriginal communities. Nutrition & dietetics: the journal of the dietitian’s association of Australia. 01 Nov 2018;75(5):448-456 | Wrong patient population |
| Taylor et al 2019 | Beyond enrolments: a systematic review exploring the factors affecting the retention of Aboriginal and Torres Strait Islander health students in the tertiary education system. Int J Equity Health. Sep 2 2019;18(1):136 | Wrong study design |
| Taylor 2019 | Creating connections: an investigation into the first year experience of undergraduate nursing students. 2009;(Ph.D.):N.PAG p-N.PAG p | Wrong setting |
| Thackrah et al 2017 | Up close and real: living and learning in a remote community builds students’ cultural capabilities and understand of health disparities. International Journal for Equity in Health. 2017;16(1) | Wrong study design |
| Thackrah and Thompson 2019 | Learning from follow-up of student placements in a remote community: a small qualitative study highlights personal workforce benefits and opportunities. BMC Medical Education. 04 Sep 2019;19(1):331 | Wrong patient population |
| Thackrah and Thompson 2013 | Confronting uncomfortable truths: receptivity and resistance to Aboriginal content in midwifery education. Contemporary Nurse. Dec 2013;46(1):113-122 | Wrong setting |
| Thackrah and Thompson 2018 | Applying a midwifery lens to indigenous health care delivery: the contribution of campus learning and rural placements to effecting systemic change. The Canadian Journal of nursing research. 01 Dec 2018;50(4):179-188 | Wrong study design |
| Thackrah et al 2015 | Promoting women’s health in remote Aboriginal settings: Midwifery students’ insights for practice. The Australian Journal of Rural Health. 01 Dec 2015;23(6):327-331 | Wrong study design |
| Thackrah et al 2014 | “Listening to the silence quietly”: investigating the value of cultural immersion and remote experiential learning in preparing midwifery students for clinical practice. BMC Research Notes. 2014;7():685 | Wrong study design |
| Thackrah et al 2015 | Exploring undergraduate midwifery students’ readiness to deliver culturally secure care for pregnant and birthing Aboriginal women. BMC Medical Education. 2015;15():77 | Wrong setting |
| Thackrah et al 2020 | Cultural respect in midwifery service provision for Aboriginal women: longitudinal follow-up reveals the enduring legacy of targeted program initiatives. International Journal for Equity in Health. 2020;19(1) | Wrong patient population |
| Thibeault 2019 | Understanding Indigenous culture through service learning and cultural immersion. Journal of Social Work Education. 2019;55(3):476-488 | Wrong study design |
| Thompson et al 2021 | The significance of Indigenous field education: an analysis of the social workers advancing through grounded education program. Journal of Human Rights and Social Work. ():1-12 | Wrong setting |
| Turale and Miller 2006 | Improving the health of indigenous Australians: reforms in nursing education. An opinion piece of international interest. Int Nurs Rev. Sep 2006;53(3):171-7 | Wrong study design |
| Turner 2005 | Awareness gained from rural experience: A student’s perspective. Australian Journal of Rural Health. August 2005;13(4):258 | Wrong study design |
| Usher et al 2005 | An innovative nurse education program in the Torres Strait Islands. Nurse Educ Today. Aug 2005;25(6):437-41 | Wrong outcomes |
| Vass and Adams 2020 | Teaching Indigenous health: racism, white privilege, and cultural self-reflexivity. Australian Journal of Primary Health. 2020;26(4):lvi-lvii | Wrong patient population |
| Vass and Adams 2021 | Educator perceptions on teaching indigenous health: racism, privilege and self-reflexivity. Medical Education. 01 Feb 2021;55(2):213-221 | Duplicate |
| Vass et al 2019 | A qualitative study exploring educator perceptions on teaching racism, privilege and cultural self-reflexivity within a general practice teaching program. Australian Journal of primary Health. 2019;25(3):liii | Wrong patient population |
| Vaughan et al 2018 | ‘Hey, we are the best ones at dealing with our own’: embedding a culturally competent program for Maori and Pacific Island children into a mainstream health service in Queensland, Australia. Journal of racial and ethnic health disparities. 01 Jun 2018;5(3):605-616 | Wrong patient population |
| Veale 2010 | Indigenous & remote health. Nurses make the move to remote communities. Australian Nursing Journal. 2010;17(8):42-42 | Wrong study design |
| Veenema 2001 | An evidence-based curriculum to prepare students for global nursing practice. Nursing and health care perspectives. Nov/Dec 2001;22(6):292-298 | Wrong setting |
| Venville and Andrews 2020 | Building great health care teams: enhancing interprofessional work readiness skills, knowledge and values for undergraduate health care students. Journal of interprofessional care. 01 Mar 2020;34(2):272-275 | Wrong outcomes |
| Vidyarthi et al 2017 | Hot in the tropics. J Hosp Med. Jun 2017;12(6):462-466 | Wrong study design |
| Virdun et al 2013 | Working together to make indigenous health care curricula everybody’s business: a graduate attribute teaching innovation report. Contemporary Nurse. Dec 2013;46(1):97-104 | Wrong study design |
| Wain et al 2016 | Engaging Australian aboriginal narratives to challenge attitudes and create empathy in health care: a methodological perspective. BMC Medical Education. 02 Jun 2016;16():156 | Wrong patient population |
| Walt and Smith 1980 | From the classroom to the community: teaching primary health workers. Journal of tropical medicine & hygiene. Aug 1980;83(4):161-4 | Wrong setting |
| Wand 2009 | Undertaking a fellowship in indigenous mental health. Australian Psychiatry. 2009;17(SUPPL. 1): S96-S99 | Wrong study design |
| Webster 2010 | Teaching medical students about aboriginal health: the carrots and the sticks. Internal Medicine Journal. March 2010;40():61 | Wrong study design |
| Webster et al 2011 | Safeguard or mollycoddle? Medical student placements in aboriginal communities. Medical Journal of Australia. 17 Oct 2011;195(8):449-450 | Wrong study design |
| Webster et al 2011 | Safeguard or mollycoddle? Medical student placements in aboriginal communities. Medical Journal of Australia. 2011;195():449-450 | Duplicate |
| West et al 2021 | Effect of a culturally safe student placement on students’ understanding of, and confidence with, providing culturally safe podiatry care. Journal of Foot and Ankle Research. Jan 2021;14(1)():9 | Duplicate |
| West et al 2016 | Culturally capable and culturally safe caseload care for indigenous women by indigenous midwifery student. Women and Birth. 01 Dec 2016;29(6):524-530 | Wrong patient population |
| West et al 2018 | Validation of the first peoples cultural capability measurement tool with undergraduate health students: a descriptive cohort study. Nurse Education Today. 01 May 2018;64():166-171 | Wrong setting |
| West et al 2013 | Indigenous Australians’ participation in pre-registration tertiary nursing courses: a mixed methods study. Contemporary Nurse. Dec 2013;46(1):123-134 | Wrong patient population |
| West et al 2017 | Development of a first peoples-led cultural capability measurement tool: a pilot study with midwifery students. Women and birth. June 2017;30(3):236-244 | Wrong setting |
| West et al 2021 | Measuring effectiveness of cultural safety education in First Peoples health in university and health services. Contemporary Nurse. 57(5):356-369 | Wrong setting |
| West et al 2016 | Creating walking tracks to success: A narrative analysis of Australian aboriginal and Torres Strait Islander nursing students’ stories of success. Collegian. 2016;23(4):349-354 | Wrong patient population |
| Wilkie et al 2023 | Indigenous Model to Support Nursing Student Success, Cultural Identity and Workforce Diversity. The Journal of nursing education 2023 Vol. 62(9) Pages 523-527 | Wrong outcomes |
| Wilkinson et al 1999 | Establishing a new university department of rural health: the first 2 years of the South Australian centre for rural and remote health. The Australian journal of rural health. Nov 1999;7(4):223-228 | Wrong outcomes |
| Williams et al 2021 | A case mix study of patients seen within an urban Aboriginal Health Service dermatology clinic over a five-year period. Australasian Journal of Dermatology. Aug 2021;62(3)():331-335 | Wrong outcomes |
| Williamson et al 2018 | Midwifery education: understanding the impact of colonisation on the wellbeing of the first people of Australia. Women and Birth. October 2018;31(Supp 1):S19 | Wrong setting |
| Williamson et al 2018 | Midwifery education: understanding the impact of colonisation on the wellbeing of the first people of Australia. Women and Birth. October 2018;31(Supp 1):S19 | Wrong setting |
| Wilson et al 2020 | Action research to implement an indigenous health curriculum framework. Nurse education today. 06 May 2020;91():104464 | Wrong setting |
| Winsor 2011 | Safeguard or mollycoddle? Medical student placements in aboriginal communities. Medical Journal of Australia. July 2011;195(2):102-104 | Wrong study design |
| Winsor 2011 | Safeguard or mollycoddle? Medical student placements in aboriginal communities. Medical Journal of Australia. 2011;194(10):497-500 | Duplicate |
| Withall et al 2021 | Assessing cultural safety in Aboriginal and Torres Strait Islander health. The Australian journal of rural heath. 2021;01 | Wrong patient population |
| Withall et al 2020 | Assessing cultural safety in Aboriginal and Torres Strait Islander Health. Australian Journal of Rural Health. 29(2):201-210 | Wrong patient population |
| Wolfgang 2011 | Change in occupational therapy students’ knowledge and attitudes towards indigenous culture after attending an indigenous cultural excursion. Australian occupational therapy journal. 2011;58:88-88 | Wrong outcomes |
| Woolley et al 2017 | Predictors of remote practice location in the first seven cohorts of James cook university MBBS graduates. Rural & remote health. 2017;17:1-11 | Wrong patient population |
| Woolley et al 2013 | Indigenous perspectives on the desired attributes of medical graduates practicing in remote communities: a northwest Queensland pilot study. The Australian journal of rural heath. Apr 2013;21(2):90-96 | Wrong patient population |
| Worley et al 2019 | The northern territory medical program – growing our own in the NT. Rural and remote health. 01 May 2019;19(2):4671 | Wrong outcomes |
| Yeoh et al 2021 | Impact of clinical placement sites on general practice as a career preference for Australian medical students. Australian Journal of Rural Heath. 30(1):95-102 | Wrong outcomes |
| Zhou et al 2012 | Efficacy of a 3-hour Aboriginal health teaching in the medical curriculum: are we changing student knowledge and attitudes? Health education journal. 2012;71(2):180-188 | Wrong setting |
| Zimmerman et al 2019 | Review of aboriginal and Torres strait islander content withing a Bachelor of Nursing. Collegian. 2019;26(4):441-447 | Wrong patient population |
| Australian Nursing Journal 2004 | Working life. Respect and reconciliation. Australian Nursing Journal. 2004;11(10):27-27 | Wrong study design |
| Australian Nursing Journal 2009 | Working Life. Australian Nursing Journal. 2009; 16(7):26-26 | Wrong study design |
| Indigenous nursing experts 2018 | The long history of indigenous nursing. Lamp. 2018;75(6):24-25 | Wrong study design |
| Contemporary Nurse: A Journal for the Australian Nursing Profession 1998 | The importance of clinical experience in Aboriginal communities. Contemporary Nurse: A Journal for the Australian Nursing Profession. 1998;7(3):152-155 | Wrong study design |
| Australian Doctor Group 2009 | Remote placement ignites a spark. Pharmacy News. 2009;():14-14 | Wrong study design |
| Australian Doctor Group 2011 | Bush incentives a mystery. Pharmacy News. 2011;():11-11 | Full text not available |
| Australian Nursing Journal 2007 | An Indigenous student nurse’s experience. Australian Nursing Journal. 2007;14(7):24-24 | Wrong study design |
| Health Promotion: Global Perspectives 2001 | A graduate diploma in indigenous health promotion in Sydney, Australia. Michigan American Journal of Health Promotion 2001;3(6):3-4 | Full text not available |
