# Supplementary material for: Determining health professional students’ self‐perceived cultural capability following participation in clinical placement with Aboriginal and Torres Strait Islander Peoples: A systematic review
Source: J Foot Ankle Res. 2024 Dec 9;17(4):e70017. doi: 10.1002/jfa2.70017 (PMC11628354; doi:10.1002/jfa2.70017)
Supplement: Supplementary file 2 — Supporting Information S2 [file JFA2-17-e70017-s003.docx]

| **Supplementary File 3:** Aboriginal and Torres Strait Islander Quality Appraisal Tool | | | | | | | | | | | | | | |
| --- | --- | --- | --- | --- | --- | --- | --- | --- | --- | --- | --- | --- | --- | --- |
|  | Askew 2017 [21] | Bennet 2013 [26] | Benson 2015 [27] | Bird 2022 [25] | Jamrozik 1995 [18] | Kamien 1975 [22] | Lockhart 2003 [23] | Morrissey 2014 [28] | Paul 2006 [12] | Power 2020 [24] | Warren 2016 [19] | Webster 2010 [29] | West 2021 [20] | Wright 2014 [30] |
| 1. Did the research respond to a need or priority determined by the community? | Y | P | Y | Y | U | U | U | U | U | U | U | U | Y | U |
| 2. Was community consultation and engagement appropriately inclusive? | Y | U | Y | Y | U | U | U | U | U | U | U | U | Y | U |
| 3. Did the research have Aboriginal and Torres Strait Islander research leadership? | U | U | Y | Y | U | N | U | N | Y | U | U | N | Y | U |
| 4. Did the research have Aboriginal and Torres Strait Islander governance? | Y | U | U | Y | U | N | U | U | U | U | Y | U | Y | U |
| 5. Were local community protocols respected and followed? | Y | U | Y | Y | U | U | U | U | U | U | U | U | Y | U |
| 6. Did the researchers negotiate agreements in regard to rights of access to Aboriginal and Torres Strait Islander peoples’ existing intellectual and cultural property? | U | U | U | U | U | N | U | U | U | U | U | U | P | U |
| 7. Did the researchers negotiate agreements to protect Aboriginal and Torres Strait Islander peoples' ownership of intellectual and cultural property created through the research? | U | U | U | U | U | N | U | U | U | U | U | U | U | U |
| 8. Did Aboriginal and Torres Strait Islander peoples and communities have control over the collection and management of research materials? | U | U | U | U | U | N | N | U | U | U | U | U | U | U |
| 9. Was the research guided by an Indigenous research paradigm? | N | N | N | P | N | N | N | P | P | N | N | N | P | N |
| 10. Does the research take a strengths-based approach, acknowledging and moving beyond practices that have harmed Aboriginal and Torres Strait peoples in the past? | Y | Y | Y | Y | P | P | U | U | U | P | U | U | Y | U |
| 11. Did the researchers plan to and translate the findings into sustainable changes in policy and/or practice? | Y | Y | P | Y | P | U | U | U | Y | P | U | U | U | U |
| 12. Did the research benefit the participants and Aboriginal and Torres Strait Islander communities? | Y | Y | Y | Y | U | U | U | U | U | U | U | U | P | U |
| 13. Did the research demonstrate capacity strengthening for Aboriginal and Torres Strait Islander individuals? | U | U | U | U | P | N | U | U | U | P | U | U | P | U |
| 14. Did everyone involved in the research have opportunities to learn from each other? | Y | U | Y | Y | p | U | U | U | U | P | U | U | Y | U |
|  | | | | | | | | | | | | | | |

Y = yes; P = partially; N = no; U = Unclear
